# Volatile Dynamics and Their Associations with Microbial Genera in Spanish-Style Table Olives Processed in Interconnected Under-Vacuum and Fiberglass Independent Vessels

**DOI:** 10.3390/foods15173034

**Published:** 2026-08-27

**Authors:** Gjergj Lekocaj, Amparo Cortés-Delgado, Alfredo Montaño, Elio López-García, Juan José Monis-Vidarte, Virginia Martín-Arranz, Francisco Noé Arroyo-López, Antonio Benítez-Cabello, Antonio Garrido-Fernández

**Affiliations:** 1 Oleica Start-Up (Technological Applications for Improvement of the Quality and Safety in Foods), Avenida Diego Martínez Barrio Nº 10, Second Floor, 41013 Seville, Spain; g.lekocaj@oleica.es; 2 Food Biotechnology Department, Instituto de la Grasa (CSIC), Campus Universitario Pablo de Olavide, Ctra. Sevilla-Utrera, km 1, Building 46, 41013 Seville, Spain; acortes@ig.csic.es (A.C.-D.); amontano@ig.csic.es (A.M.); elopez@ig.csic.es (E.L.-G.); jj.monis@ig.csic.es (J.J.M.-V.); v.martin@ig.csic.es (V.M.-A.); fnoe@ig.csic.es (F.N.A.-L.); agarrido@ig.csic.es (A.G.-F.)

**Keywords:** table olives, Spanish-style, volatile organic compounds (VOCs), fermentation, metataxonomy

## Abstract

This study compared the volatile organic compound (VOC) profiles and their relationships with microbial genera in Spanish-style table olives fermented in an interconnected under-vacuum stainless-steel system (F) and in traditional independent fiberglass vessels (G). VOCs were analyzed by HS-SPME-GC-MS, and microbial communities were characterized by metataxonomic analysis. A total of 97 VOCs were identified, mainly alcohols (29), esters (21), carbonyls (20), hydrocarbons (5), phenols (4), and terpenes (6). Among the most abundant compounds were 4-ethylphenol, ethanol, (Z)-3-hexen-1-ol, phenylethyl alcohol, ethyl acetate, and isobutanol. Only five VOCs—decanal, methyl ethyl ether, linalool, 2-methylbutanoic acid, and ethyl lactate—showed no significant variation with fermentation system or time, suggesting formation during debittering or early fermentation. The fermentation system strongly influenced volatile development, with 16 VOCs unique to F, 15 unique to G, and 66 shared. Among shared compounds, 48 differed significantly between systems, and 43 changed during fermentation. The F system was associated with a more restricted VOC profile, whereas G generated a more diverse volatile profile. Microbial–VOC relationships also differed: *Leuconostoc*, *Lactiplantibacillus*, *Candida*, and *Dekkera* were mainly associated with F-system VOCs, whereas less common bacterial genera and fewer fungi characterized G. Overall, the interconnected under-vacuum system is suitable for standardized production, while the traditional system favors greater microbial and volatile complexity.

## 1. Introduction

According to the Trade Standard Applying to Table Olives, table olives are produced from sound fruits of selected *Olea europaea* L. varieties suitable for processing and consumption [1]. The earliest known description of their preparation appears in *De Re Rustica* [2]. Because olives are naturally bitter, a range of debittering techniques has been developed over time, with alkaline mineral ashes proving especially effective. This practice ultimately evolved into the modern green Spanish-style process, which uses sodium hydroxide solutions. Similar alkaline treatments were also used elsewhere in the Mediterranean; for example, several authors report their use in Italy in the early 19th century [3,4].

During the first half of the 20th century, lye treatment and washing were commonly performed in cement tanks, while fermentation took place in traditional wooden barrels (*bocoyes*) made of oak or chestnut, each holding about 800 L. Smaller 130-L barrels were used for export, with retail packaging often completed in destination markets such as the United States and Canada [5]. After visiting Spain and Greece, the University of California at Davis (USA) published “*Pickling Green Olives*” [6], initiating a line of research that later produced “*Production of Spanish-type green olives*” [7]. This work represents the first attempt to perform lye treatment, washing, and fermentation in a single large container, using wooden or cement tanks of about 7000 kg [8].

In Spain, systematic research advanced with the creation of the Instituto de la Grasa (Sevilla), whose results were compiled in “*El aderezo de aceitunas verdes*” [9]. By the 1960s, Spain exported about 40,000 tons of table olives annually [5], yet the production process had changed little. Its expansion was constrained by high labor requirements, the high cost and sanitizing challenges of wooden barrels, difficulties in controlling fermentation and bulk storage, and the need for frequent brine replenishment due to brine losses [10]. In contrast, Argentina adopted quadrangular cement tanks with plastic-coated interiors (3000–4000 L) for both lye treatment and fermentation [11], while Greece employed fermenters similar to those used for naturally black olives [12].

Spanish *bocoyes* were replaced in the mid-1960s by fiberglass fermenters (1800–10,000 L), which facilitate handling of olives and brine and were soon adopted in other producing countries [10,13]. Although the process has since become largely standardized, it still faces limitations, including gas accumulation, overflow at the onset of fermentation, uncontrolled microbial dynamics, and product variability. To address some of these challenges, a promising innovation is the recently tested interconnected under-vacuum fermentation system, which stabilizes physicochemical and microbiological parameters, resulting in more predictable quality outcomes [14].

A defining characteristic of Spanish-style green olives is their aroma and flavor, closely tied to their volatile organic compound (VOC) profile. A study compared *Nocellara del Belice* olives processed using Spanish-, Greek-, and Castelvetrano-style methods, identifying 22 VOCs across major chemical groups and demonstrating that processing technology strongly influences VOC composition and, consequently, olive flavor [15]. With advances in GC-MS, the number of reported VOCs has increased considerably. A total of 102 VOCs was identified in *Manzanilla*, *Gordal*, and *Hojiblanca* olives, and PCA revealed cultivar-related differences; only (E)-2-decenal showed statistically significant differences among cultivars [16]. Agronomic factors also play a role: regulated deficit irrigation (RDI) modified the VOC profile of Spanish-style *Manzanilla* olives, increasing alcohols, ketones, and phenolic compounds while reducing esters and organic acids. These shifts enhanced green-olive flavor and decreased bitterness, supporting the potential of *hydroSOStainable* olives [17].

Microbiota activity further shapes VOC formation. In Sicilian table olives, it was found that *Lactiplantibacillus plantarum* suppressed the formation of 4-ethylphenol, derived from *p*-cumaric acid, thereby minimizing phenolic off-flavor. However, the compound eventually appeared in control samples by day 80. Nevertheless, VOC profiles showed no major qualitative differences across fermentations, leading the authors to conclude that the cultivar exerted a stronger influence than the microbiota [18]. In contrast, distinct VOC profiles were observed when mixed starter cultures (*L*. *pentosus* plus *Wickerhamomyces anomalus*) were applied. Some compounds (e.g., 2-phenylethyl acetate and cis-2-penten-1-ol) were common to all cultures, whereas others (e.g., methanol and 4-ethylphenol) were specific to strain Lp115. Additional compounds—including 4-ethylphenol (Lp115), 5-tert-butylpyrogallol (Lp13, Lp115), and 2-phenyl ethanol (Lp13)—showed strain-specific inhibition [19]. These findings indicate that process adjustments, particularly in starter-culture selection, can substantially affect VOC development.

Building on this foundation, the present study compares the volatile profile of Spanish-style green table olives fermented in a newly developed interconnected under-vacuum stainless-steel system with that of olives fermented using a traditional process in independent fiberglass fermenters. Both systems had comparable working capacities and were inoculated with the same starter culture. The relationship between VOC profiles and microbial taxa detected at the end of fermentation was also examined.

## 2. Materials and Methods

### 2.1. Experimental Design

The study compared a newly developed, closed, interconnected, under-vacuum stainless-steel fermentation system (hereafter the F-system)—designed and assembled at the pilot plant of the Instituto de la Grasa (CSIC, Seville, Spain)—with traditional, independent fiberglass fermenters (the G-system). The F-system operates predominantly under anaerobic conditions, whereas the G-system is more aerobic, with its top opening exposed to the atmosphere.

**Figure 1:**
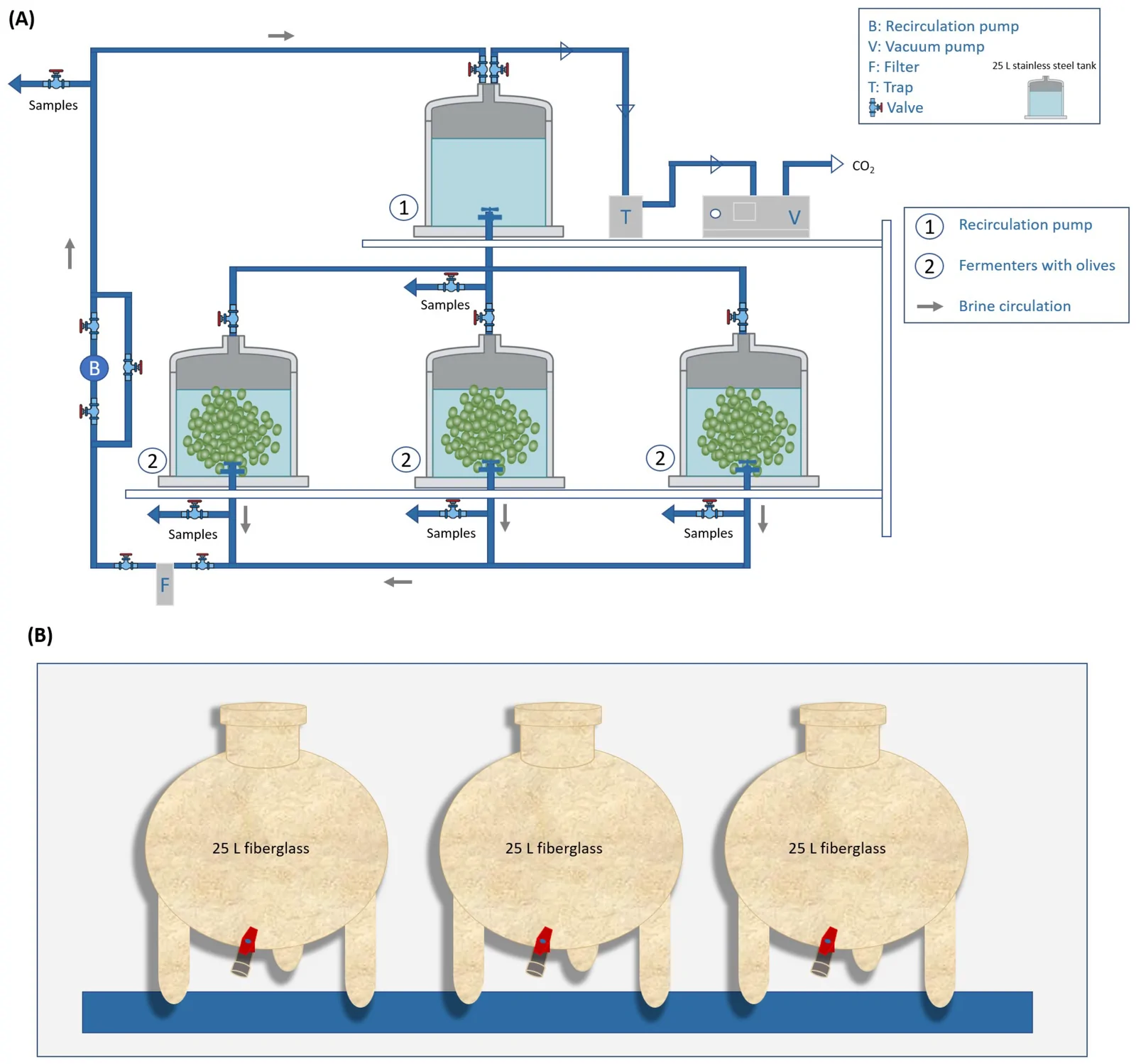
Schematic representation of the new under-vacuum-interconnected fermentation system (**A**) (System F). Redrawn from [14]. System G consisted solely of three individual spherical fiberglass vessels (**B**), as described in the text and in [14].

The F-system consisted of four interconnected 25-L stainless-steel vessels arranged in two tiers, including a vacuum-operated brine reservoir and three fermentation units. A schematic of the system is shown in Figure 1A. The system contained a total of 51.6 kg of olives (about 17.2 kg per vessel) and 45 L of brine (15 L per vessel). Brine recirculation and vacuum extraction were used to homogenize the system and remove accumulated gases. Detailed technical specifications are provided elsewhere [14].

The G-system consisted of three independent fiberglass fermenters (25 L each; approximately 1:750 scale relative to conventional industrial 16,000-L vessels) Figure 1B. To replicate standard industrial practice, the fermenters were covered with non-hermetic lids, allowing gas exchange while protecting the fermentation from external contamination. Each fermenter contained 15.04 kg of olives and 9.32 L of brine.

### 2.2. Olives, Debittering Process, and Fermentation

Manzanilla olives harvested at the green maturity stage during the 2022/2023 season were processed using the Spanish style. Debittering was performed on an industrial scale at ARASOL S.L. (Arahal, Seville, Spain). The fruits were treated with a 3.2% (*w*/*v*) NaOH solution until the lye penetrated roughly two-thirds of the flesh, followed by a 6-h wash to remove excess alkali. The olives were then transported to the Instituto de la Grasa (CSIC, Sevilla, Spain), where the wash water was drained, and the fruits were distributed among the six fermenters described above: three for the F-system and three for the G-system. Finally, the olives were covered with a 9% (*w*/*v*) NaCl brine supplemented with 3 mL of hydrochloric acid (37% purity), yielding an initial brine pH of approximately 9.3 before the onset of fermentation.

The fermenters were inoculated with the commercial starter culture Oleica Starter Advance (Oleica, Seville, Spain) 3 days after brining. Before inoculation, the lyophilized starter culture was rehydrated in the corresponding fermentation brine for 15 min. In the F-system, the starter was added exclusively to the lung vessel, and brine circulation distributed it throughout all fermenters. In the G-system, each fermenter was inoculated independently. In both systems, the starter cultures reached a final concentration of 10^6^ CFU/mL. The inoculum consisted of equal proportions of lyophilized cultures of *Lactiplantibacillus pentosus* LPG1, 13B4, and 119 strains, previously characterized for their probiotic potential and technological relevance.

### 2.3. Metataxonomic Analysis

For the metataxonomic analysis of bacterial and fungal taxa, samples were collected after 22 days of fermentation, corresponding to the active fermentation stage. The 16S DNA and ITS regions were sequenced, respectively. A detailed description of the sample processing methodology, DNA extraction and purification, and sequencing process is provided elsewhere [14], which also describes the impact of the new fermentation system on the fermentation microbiota.

### 2.4. VOCs Analysis

VOCs were analyzed by headspace solid-phase microextraction (HS-SPME) coupled with gas chromatography-mass spectrometry (GC-MS), following the method described previously [20] with slight modifications. Brine samples were collected on days 9 and 55 of fermentation from each vessel (*n* = 12 samples, analyzed in duplicate). A 2 mL aliquot of each brine sample was placed into a 15 mL glass vial, and 20 µL of internal standard (6-chloro-2-hexanone, 40 mg/L) was added. The vial was sealed and placed in a water bath (at 40 °C). Samples were equilibrated for 15 min at 40 °C with stirring (600 rpm), using a stirring bar. VOCs were extracted for 30 min on a divinylbenzene/carboxen/polydimethylsiloxane (DVB/CAR/PDMS) SPME fiber (2 cm, 50/30 µm; Supelco, Bellefonte, PA, USA). The adsorbed VOCs were then desorbed in the GC-MS injector port at 265 °C for 15 min (electron ionization: 70 eV). Chromatographic separation was performed using a VF-WAX MS capillary column (30 m, 0.25 mm, 0.25 μm film thickness; Agilent, Santa Clara, CA, USA). The oven program was: 40 °C for 5 min; 40–195 °C at 3 °C/min; then 195–240 °C at 10 °C/min, held for 15 min. Helium was used as the carrier gas at a constant flow of 1 mL/min.

Peak detection and quantification were performed using MassHunter (Agilent Technologies, Santa Clara, CA, USA) with the software deconvolution algorithm. Tentative identification of deconvoluted peaks was achieved using the NIST17MS library, accepting matches with a mass spectral similarity score >80 (100 is an exact match). Identification was further supported by comparing experimental retention indices with literature values and authentic standards. VOCs were semi-quantified by expressing their peak areas relative to the internal standard (6-chloro-2-hexanone), assuming similar response factors across compounds.

### 2.5. Statistical Analysis

The volatile data (means from two analyses of biological replicates) were treated as a two-way factorial design. Statistical analysis proceeded in several steps. Relative VOC concentrations were first evaluated using two-way ANOVA in XLSTAT v. 2017 (Addinsoft, Paris, France) and Statistics v. 8.0 (StatSoft, Inc., Tulsa, OK, USA). Main effects and interactions were considered significant at *p* < 0.05.

Column-centered data were then subjected to Multifactor analysis (MFA) using the R package *factoextra* v. 1.0.7 (13 October 2022) [21], which extracts and visualizes results from *FactoMineR* v2.11 (20 April 2024) [22]. The analyses were performed in R v. 4.5.0 [23].

Microbial community data (bacterial 16S and fungal ITS sequences) from each fermenter after 22 days of fermentation were obtained from our previous study [14]. VOC profiles were associated with microbial taxa using Pearson correlation (*p* < 0.05) and the PLS-R option in XLSTAT 2017 (Addinsoft, Paris, France). Network visualization of the VOC—taxa relationship was performed using *Cytoscape* v. 3.10.4 [24].

## 3. Results and Discussion

The physicochemical, microbiological, and sensory changes during Spanish-style olive fermentation in the new interconnected under-vacuum stainless-steel system—as compared with the traditional independent fiberglass fermenters— have already been described [14].

The synthesis of comparative results indicates that the new system reduced variability across most physicochemical and microbiological parameters, reflecting greater process uniformity. It also maintained lower CO_2_ concentrations throughout the process, produced firmer products, enabled more consistent inoculum imposition, and was associated with a lower combined acidity at the end of the fermentation. In contrast, the conventional system promoted the growth of lactic acid bacteria, resulting in higher acidity (despite similar pH), a more characteristic color, and slightly higher overall acceptability.

However, both systems received comparable sensory evaluations across most attributes. Nevertheless, further optimization and validation at the industrial scale remained essential [14]. Overall, these findings suggest that the new system provides a more robust and reproducible fermentation process while maintaining sensory quality comparable to that of the conventional system. Building on that work, the present study focuses specifically on the volatile organic compounds (VOCs) generated in both systems, comparing their compositions and examining potential relationships between these VOC profiles and their corresponding microbial populations.

The average VOC concentrations for fermenters, fermentation systems, and fermentation periods are shown in Table S1 (Supplementary Material). A total of 97 VOCs were identified, including 7 volatile acids, 29 alcohols, 20 carbonyl compounds, 21 esters, 5 hydrocarbons, 4 phenols, 6 terpenes, and 6 “other” compounds (Table S1, Supplementary Material). Most VOCs were present in both systems, although several were system-specific.

Alcohols were the most abundant chemical group, followed by phenols, esters, and “other” compounds (Table S1). ANOVA showed that the F-system produced significantly higher levels of alcohols, esters, and phenols overall. In contrast, acids, carbonyls, and hydrocarbons were significantly higher in the G-system. Acids, alcohols, esters, and terpenes increased significantly during fermentation, whereas hydrocarbons, “others” compounds, and phenols declined (Table 1). Interactions increased significantly during fermentation, regardless of the fermentation system, for alcohols and esters, whereas phenols decreased. However, the increases in both systems in acids, carbonyls, and terpenes, or the decrease of “other” compounds during fermentation, although noticeable, were not significant. Additionally, phenols decreased in the F-system but increased over time in the G-system. Overall, acids, alcohols, carbonyls, esters, and terpenes tended to accumulate as fermentation progressed, while hydrocarbons, “other” compounds, and phenols declined—suggesting that these decreasing groups likely originate from early processing steps (e.g., likely lye treatment) and are later transformed.

**Table 1:** Volatile organic compounds (VOCs) in the F and G fermentation systems. Results of the ANOVA by chemical groups, showing mean concentrations (µg/L) for fermentation system levels, fermentation periods, and their interactions, along with the corresponding *p*-values for each comparison.

Factors/Interact.			Acids	Alcohols	Carbonyls	Esters	Hydrocarbons	Others	Phenols	Terpenes
Overall model		N = 24	686 (80)	3079 (261)	134 (13)	333 (73)	86 (7)	152 (28)	889 (167)	47 (2)
		*p*-value	<0.0001	<0.0001	<0.0001	<0.0001	<0.0001	<0.0001	<0.0001	<0.0001
F-system		N = 12	567 (109)	4167 (239)	87 (6)	483 (132)	60 (7)	175 (46)	1654 (93)	44 (3)
G-system		N = 12	806 (114)	1991 (114)	182 (16)	182 (23)	112 (7)	129 (33)	124 (36)	51 (4)
		*p*-value	**<0.0001**	**<0.0001**	**<0.0001**	**<0.0001**	**<0.0001**	0.1451	**<0.0001**	0.0712
9 days		N = 12	326 (44)	2600 (250)	124 (17)	79 (9)	101 (10)	264 (32)	1009 (280)	41 (3)
55 days		N = 12	1046 (44)	3558 (426)	144 (20)	586 (102)	72 (9)	40 (2)	770 (189)	54 (3)
		*p*-value	**<0.0001**	**<0.0001**	0.2611	**<0.0001**	**0.0013**	**<0.0001**	**0.0004**	**0.0050**
F-system	9 days	N = 6	208 (10)	3408 (72)	75 (8)	50 (3)	75 (6)	308 (46)	1931 (41)	38 (1)
	55 days	N = 6	925 (24)	4926 (128)	99 (4)	916 (45)	45 (8)	42 (4)	1378 (76)	50 (3)
G-system	9 days	N = 6	445 (54)	1792 (88)	174 (16)	108 (7)	126 (10)	220 (39)	86 (49)	45 (6)
	55 days	N = 6	1167 (46)	2190 (184)	189 (30)	257 (9)	98 (6)	38 (3)	162 (52)	57 (4)
		*p*-value	0.9506	**0.0002**	0.8043	**<0.0001**	0.9042	0.1795	**<0.0001**	0.9826

Notes: Means (standard error in parentheses). F-system, fermentation under interconnected, under-vacuum stainless-steel fermenters. G-system, fermentation in traditional independent fiberglass fermenters. *n*= total number of independent values participating in the comparisons and contributing to means and standard errors.

The number of VOCs reported in table olives varies considerably across cultivars, processing conditions, and analytical methods. Previous studies have identified between 22 and 102 VOCs across different table olive preparations, with alcohols, phenols, acids, esters, aldehydes, and ketones among the predominant chemical groups. Therefore, the 97 VOCs detected in the present study and their distribution across chemical classes are consistent with previous reports.

VOCs exhibited three main temporal patterns (Figure S1): (i) compounds present from the start of fermentation and remaining relatively stable; (ii) compounds that increased during fermentation and are therefore likely fermentation-derived; and (iii) compounds that decreased over time, suggesting consumption or transformation. Several fermentation-derived VOCs, particularly esters, phenolic compounds, and terpenoid derivatives, have been reported in Spanish-style table olives and are commonly associated with microbial activity during fermentation [20,25,26,27].

Another group of VOCs decreased during fermentation (Figure S1), including several alcohols, esters, hydrocarbons, and phenolic compounds, suggesting their formation during alkaline treatment or early fermentation followed by subsequent transformation. Representative examples were theaspirane A, theaspirane B, coumaran, and 4-ethylphenol. However, although it is tempting to infer precursor-product relationships between decreasing and increasing compounds, such an interpretation requires caution, as multiple biochemical pathways may act simultaneously. Among these compounds, the hydrocarbon decane has been reported sporadically in both Spanish [16] and Greek cultivars (Kalamata, Conservolea, and Halkidiki) [28]. Theaspirane B appears only occasionally in the volatilome of Spanish-style green olives at relatively low concentrations [16,25], while coumaran is generally rare.

### 3.1. Effect of Fermentation System and Processing Period on Common Volatile Organic Compounds

Given the strong influence of design variables, VOC patterns were analyzed using ANOVA of the full data set (Table S1). Common VOCs—those detected in both fermentation systems)—are discussed separately from system-specific compounds.

Among the common compounds, four VOCs—decanal, 2,4-dimethylbenzaldehyde, methyl hexyl ether, and linalool—were unaffected by the system, fermentation time, and their interaction (Table 2), and remained at low, stable levels. Their consistent behavior suggests that they originate from the fruits or early processing stages rather than from fermentation, limiting their value as process markers. In previous studies, decanal has been reported only sporadically in Spanish-style green olives [29], whereas linalool is frequently detected but usually at similar low concentrations [25].

Several other compounds showed partial effects of the fermentation system or time. For example, 2-methylbutanoic acid was unaffected by the system alone but increased significantly over time, with a higher final level in the F-system (Table 2). This acid has been reported at low levels [16], with significant cultivar-dependent differences, and with a systematic increase during post-fermentation. Its formation has been attributed to lye treatments [29] or to the activity of the *Pichia manshurica* NC168.3 strain [20]. In the present study, 2-methylbutanoic acid could have been generated during lye treatment and throughout fermentation, particularly under F-system conditions, suggesting that its formation was not associated with *Pichia* yeast. Ethyl lactate, initially absent or present at low levels, increased significantly throughout fermentation, mainly in the F-system, indicating a clear interaction between fermentation system and time (Table 2). This behavior is consistent with the formation of ethyl lactate from ethanol and lactic acid, which are well-established products of table-olive fermentation. Ethyl lactate has been reported in Greek Spanish-style olives from various cultivars under both high- and low-salt industrial conditions [28], although its presence is inconsistent [18,30].

Many VOCs were influenced only by the fermentation system (Table 2)—including caprylic acid, 2-pentanol, 1-hexanol, (Z)-3-hexen-1-ol, 1-octanol, 2-butanone, 2-methyl butanal, acetoin, methyl 2-methylbutanoate, (Z)-3-hexenyl acetate, and geraniol—and showed no interactions. This pattern suggests they originated from lye treatment or early fermentation, or were formed and subsequently transformed during fermentation. Conversely, a few compounds—such as 3-pentanol, 1-nonanol, 1-dodecanol, and dimethyl sulfide—were also unaffected by fermentation time but exhibited significant interactions, though the changes were too small to be biologically relevant (Table 2). Further information on the effects of the fermentation system and processing period is provided in Table 2.

### 3.2. Interactions and General Trends Among Common Volatile Organic Compounds

Twenty-six VOCs showed significant system × time interactions (Table 2; Figure S2). Several compounds (the number in parentheses indicates the order in Figure S2), including acetic acid (1), 2-methylbutanoic acid (2), phenylethyl alcohol (12), methyl acetate (16), ethyl 2-methylbutanoate (18), ethyl lactate (19), ethyl hydrocinnamate (20), and creosol (24), increased throughout fermentation in both systems, though at different rates. In contrast, ethanol (3), isobutanol (5), isopentanol (7), 2-ethylhexanol (8), and ethyl acetate (17) increased primarily in the F-system, whereas coumaran (23) and 4-ethylphenol (25) declined over time. Other VOCs showed system-dependent or less consistent trends (Table 2).

Overall, the F and G-systems followed distinct dynamic trajectories, resulting in divergent final VOC profiles. Among the interacting compounds, isopentanol (7) and 4-ethylphenol (25) reached markedly higher concentrations in the F-system, suggesting their potential use as markers of this fermentation process.

Eighteen VOCs were among the most abundant overall (Table 2). Ethanol, isobutanol, phenylethyl alcohol, ethyl acetate, creosol, and 2-methylbutanoic acid generally increased during fermentation, whereas coumaran (23) and 4-ethylphenol (25) reached maximum levels at day 9 and subsequently declined. Together, these trends indicate that lactic acid fermentation was accompanied by extensive alcohol production and subsequent ester formation, particularly in the F-system.

The most abundant VOCs detected in both systems are consistent with those previously reported for Spanish-style table olives and are widely associated with olive fermentation microbiota [27,28,31].

### 3.3. Trends in System-Specific Volatile Organic Compounds

Most VOCs unique to the F-system (Table 3, F-system) appeared only after 55 days of fermentation, indicating they formed under the interconnected, under-vacuum conditions characteristic of this process. Their concentrations were generally low, consistent with formation from minor precursors or specific microorganisms. Only three compounds were detected at days 9 and 55: octanal (which showed no significant changes), 3-methylpentanol—not previously associated with table olives— and isopentyl acetate (both showing significant increases over fermentation time (Table 3)). Among all F–specific VOCs, isopentyl acetate was the only compound to show a clear, steady rise throughout fermentation. It has also been detected in a natural olive-derived culture medium [20]. Although generally present at low concentrations, these VOCs clearly characterize the new F process.

VOCs exclusive to the G-system (Table 3, G-system) were also mostly low in concentration and showed limited evolution. Compounds such as o-guaiacol (one of the most abundant VOCs) and phenol (which was consistently low) were not consistently detected. They did not increase significantly over time, suggesting they originated primarily from lye treatment. In the literature, o-guaiacol has been reported only occasionally [19], whereas phenol is frequently described, especially in Spanish cultivars [16,29]. Conversely, 4-methyl-3-pentene-2-one, although initially present at relatively moderate levels, increased significantly during fermentation and is not commonly reported in the table olive studies. Other compounds were detected only at day 55, including acetophenone—detected at low concentrations and rarely reported in Spanish-style green olives [20]—methyl lactate, which was moderately abundant and is frequently observed in previous studies [29], and dihydroedulan II, which have been sporadically reported in Spanish-style green olives [16,29]; their late appearance strongly suggests fermentation-derived formation. Despite their generally low abundance, these G–specific VOCs help to characterize the traditional, independent G process.

### 3.4. Multivariate Analysis

Given the complexity of VOC analysis, multivariate statistical methods are particularly well suited for interpretation. In this study, a multifactor analysis (MFA) was conducted using only VOCs shared between the two fermentation systems. The fermentation system and sampling period were included as supplementary qualitative variables. System-specific compounds were excluded because their presence is inherently tied to each system and does not require further validation.

**Figure 2:**
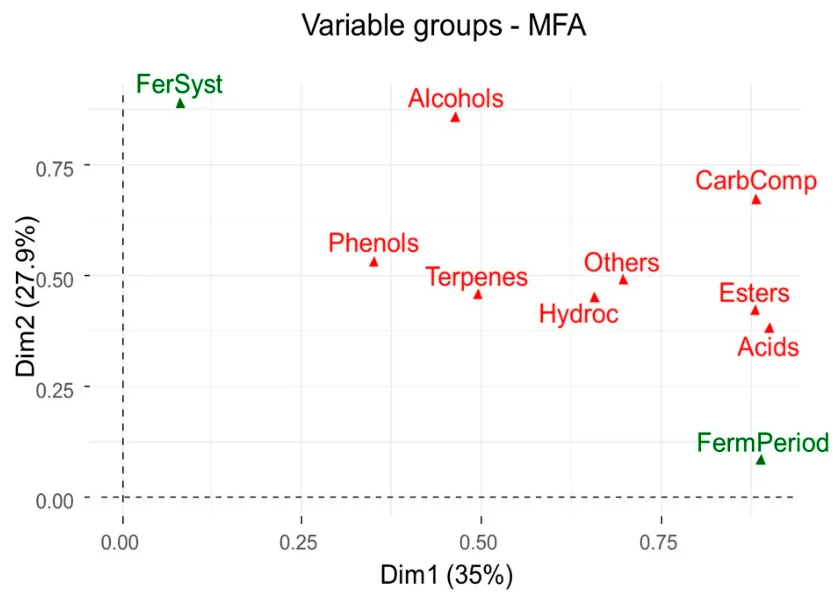
Multifactor analysis (MFA) of quantitative variable groups (volatile organic compounds, VOCs), with fermentation system (FerSyst) and sampling moment (FermPeriod) as supplementary qualitative variables—projection onto the Dim1-Dim2 plane for VOC groups, fermentation system, and sampling time. CarbComp, carbonyl compounds. Hydroc, hydrocarbons.

MFA was used to explore relationships among VOCs, the fermentation system, and the fermentation period. Projection of design variables and chemical groups onto the first two dimensions (Figure 2) showed that Dim1 was mainly associated with the fermentation period and with acids, esters, carbonyl compounds, hydrocarbons, and other compounds, whereas Dim2 was primarily related to the fermentation system and alcohols. Phenols and terpenes contributed to both dimensions. These results indicate that fermentation time mainly influenced acids, carbonyls, and esters, whereas alcohols were more strongly associated with the fermentation system.

**Figure 3:**
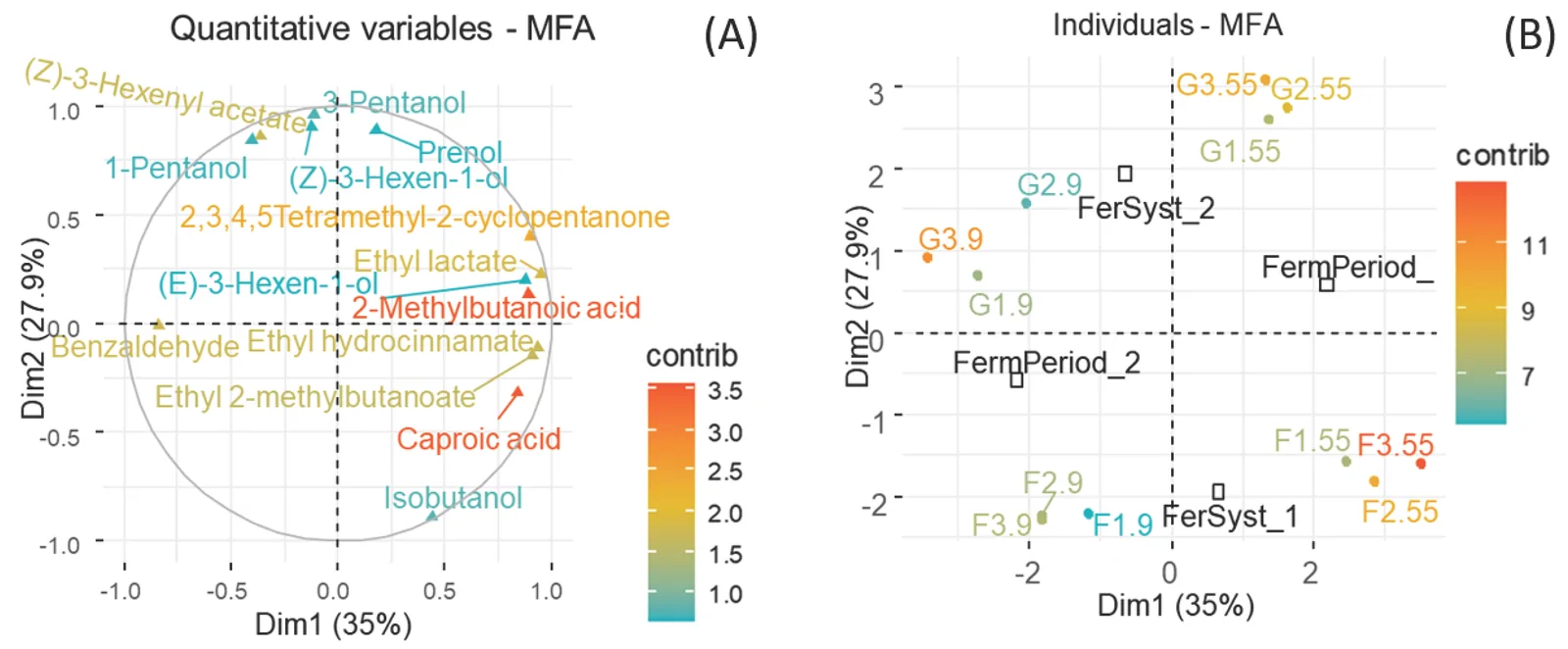
Results of the multifactor analysis of quantitative variables (VOCs), with the fermentation system and sampling moment (FermPeriod) as supplementary qualitative variables. Projection onto the Dim1-Dim2 plane, based on contributions, of (**A**) the most contributive quantitative variables, and (**B**) individuals (fermenters, G and F systems) and fermentation periods (FermPeriod). Numbers following F and G indicate the replicate number of the system and the sampling date, respectively.

The projection of individual VOCs and samples (Figure 3A,B) revealed clear separation by both fermentation period and fermentation system. Positive contributors to Dim1 included 2-methylbutanoic acid, caproic acid, ethyl lactate, ethyl hydrocinnamate, ethyl 2-methylbutanoate, and 2,3,4,5-tetramethyl-2-cyclopentanone, whereas benzaldehyde was negatively associated with this dimension, indicating its predominance during the early stages of fermentation. Dim2 mainly discriminated between the fermentation systems, with compounds such as 3-pentanol, prenol, (Z)-3-hexenyl butanoate, and 1-pentanol associated with the G-system, whereas benzaldehyde, ethyl hydrocinnamate, ethyl 2-methylbutanoate, caproic acid, and isobutanol were more closely related to the F-system.

Overall, the number of VOCs distinguishing the two fermentation systems was relatively small compared with the large set of compounds they shared. Most VOCs associated with the 55-day samples were fermentation-derived and accumulated over time, whereas benzaldehyde was the only VOC consistently associated with the early fermentation stage. Similar multivariate analyses have successfully discriminated among cultivars, geographical origins, fermentation conditions, and starter-culture effects in table olives [18].

**Table 2:** Volatile organic compounds (VOCs) common to the F and G fermentation systems. Results of the ANOVA showing mean concentration (µg/L) for fermentation system levels, fermentation periods, and their interaction, along with the corresponding *p*-values for each comparison.

Effect/Interaction	Fermentation System			Fermentation Period			FermentationSystem × Fermentation Period (Interaction)				
Factor Level	F-System	G-System	*p*-Value	9 Days	55 Days	*p*-Value	F-System	F-System	G-System	G-System	*p*-Value
							9 Days	55 Days	9 Days	55 Days	
N	12	12		12	12		6	6	6	6	
***Acids***											
** Acetic acid**	402.0 (87)	652 (102)	**<0.0001**	221 (38)	833 (48)	**<0.0001**	116 (7)	688 (11)	327 (42)	978 (42)	**<0.0001**
** 2-Methylbutanoic acid**	140.0 (20)	130.1 (11.6)	0.3526	89.5 (7.8)	180.5 (9.5)	**<0.0001**	78.1 (2.3)	201.8 (13.1)	101.0 (14)	159.3 (6.4)	**0.0048**
** Caproic acid**	14.8 (2.0)	8.5 (1.6)	**0.0019**	7.4 (1.2)	15.9 (1.9)	**<0.0001**	9.1 (0.7)	20.5 (1.8)	5.7 (2.2)	11.4 (1.9)	0.1110
** Caprylic acid**	4.7 (0.4)	1.9 (0.6)	**0.0011**	3.2 (0.7)	3.4(0.6)	0.8664	5.2 (0.5)	4.3 (0.6)	1.3 (0.8)	2.4 (1.0)	0.1756
***Alcohols***											
** Ethanol**	980.4 (85.2)	334.1 (11.5)	**<0.0001**	525.4 (56.0)	789.1 (141.7)	**<0.0001**	707.0 (21.1)	1253.9 (39.8)	343.8 (13.8)	324.3 (18.9)	**<0.0001**
** 2-Butanol**	7.7 (0.9)	13.6 (2.2)	**0.0005**	6.4 (0.6)	14.9 (1.8)	**<0.0001**	4.8 (0.3)	10.7 (0.2)	8.0 (0.5)	19.2 (2.8)	0.0728
** 1-Propanol**	21.4 2.0)	16.1 (0.8)	**0.0006**	16.6 (0.8)	21.0 (2.1)	**0.0027**	15.7 (1.0)	27.1 (1.8)	17.4 (1.2)	14.9 (1.0)	**<0.0001**
** Isobutanol**	369.7 (21.1)	8.7 (0.9)	**<0.0001**	157.9 (45.3)	220.6 (64.3)	**<0.0001**	307.0 (12.0)	432.5 (15.5)	8.8 (1.5)	8.7 (1.1)	**<0.0001**
** 3-Pentanol**	7.7 (0.3)	14.8 (0.7)	**<0.0001**	10.7 (1.0)	11.8 (1.4)	0.1019	8.0 (0.5)	7.4 (0.3)	13.3 (1.0)	16.2 (0.7)	**0.0184**
** 2-Pentanol**	12.4 (0.4)	16.1 (1.3)	**0.0080**	13.3 (0.5)	15.2 (1.4)	0.1391	12.4 (0.6)	12.4 (0.6)	14.1 (0.8)	18.0 (2.2)	0.1387
** Isopentanol**	1341.2 (46.8)	134.0 (8.9)	**<0.0001**	669.8 (164.7)	805.4 (201.3)	**0.0002**	1215.1 (19.4)	1467.2 (53.7)	124.5 (7.4)	143.5 (16.0)	**0.0009**
** 3-Methyl-3-buten-1-ol**	8.5 (0.9)	12.7 (1.3)	**0.0003**	7.7 (0.6)	13.6 (1.1)	**<0.0001**	5.6 (0.2)	11.4 (0.4)	9.7 (0.5)	15.7 (1.8)	0.9007
** 1-Pentanol**	3.5 (0.1)	7.0 (0.3)	**<0.0001**	5.5 (0.6)	4.9 (0.5)	**0.0350**	3.7 (0.2)	3.3 (0.1)	7.4 (0.3)	6.6 (0.4)	0.4956
** 4-Penten-1-ol**	2.0 (0.4)	4.1 (0.2)	**<0.0001**	3.6 (0.3)	2.5 (0.4)	**0.0015**	2.9 (0.4)	1.2 (0.4)	4.4 (0.2)	3.8 (0.2)	0.0845
** Prenol**	13.8 (0.9)	27.9 (2.2)	**<0.0001**	17.1 (1.9)	24.6 (2.9)	**0.0003**	11.2 (0.5)	16.3 (0.7)	23.0 (1.2)	32.8 (3.1)	0.1851
** 1-Hexanol**	68.1 (3.4)	111.8 (5.5)	**<0.0001**	95.1 (5.5)	84.9 (9.6)	0.1037	78.1 (2.5)	58.2 (2.2)	112.1 (3.6)	111.6 (10.9)	0.1216
** (E)-3-Hexen-1-ol**	6.3 (0.6)	5.9 (0.7)	0.4543	4.3 (0.2)	7.9 (0.4)	**<0.0001**	4.5 (0.3)	8.0 (0.2)	4.1 (0.3)	7.7 (0.7)	0.8704
** (Z)-3-Hexen-1-ol**	478.4 (11.2)	783.8 (33.5)	**<0.0001**	609.9 (40.8)	652.3 (61.1)	0.2121	488.8 (16.4)	468.1 (15.7)	731.0 (34.6)	836.5 (51.2)	0.0698
** 5-Hexen-1-ol**	4.3 (0.4)	8.4 (0.4)	**<0.0001**	7.5 (0.6)	5.2 (0.7)	**<0.0001**	5.6 (0.2)	3.0 (0.1)	9.3 (0.4)	7.4 (0.4)	0.3203
** 2-Ethylhexanol**	154.4 (12.5)	22.3 (1.1)	**<0.0001**	68.5 (14.3)	108.2 (26.0)	**<0.0001**	115.1 (4.7)	193.7 (6.5)	21.9 (1.8)	22.7 (1.6)	**<0.0001**
** 6-Hepten-1-ol**	11.2 (11.7)	42.3 (10.2)	**<0.0001**	31.0 (10.5)	22.4 (24.8)	**0.0095**	22.4 (1.3)	N/D	397 (8.0)	44.9 (12.3)	***0.0002***
** 1-Octanol**	17.5 (0.6)	24.3 (0.9)	**<0.0001**	20.5 (1.4)	21.3 (1.2)	0.4937	16.6 (0.8)	18.3 (0.9)	24.4 (1.3)	24.3 (1.3)	0.4499
** 3-Cyclopentyl-1-** ** propanol**	4.42 (0.2)	5.9 (1.3)	0.2071	6.5 (0.6)	3.8 (1.1)	**0.0233**	4.8 (0.2)	4.1 (0.2)	8.3 (0.3)	3.5 (2.2)	0.0837
** 1-Nonanol**	8.1 (0.5)	10.6 (0.7)	**0.0017**	10.0 (0.9)	8.7 (0.4)	0.0874	7.4 (0.6)	8.8 (0.8)	12.5 (0.9)	8.7 (0.5)	**0.0013**
** 1-Decanol**	3.1 (0.3)	1.4 (0.5)	**<0.0001**	3.2 (0.3)	1.3 (0.4)	**<0.0001**	3.5 (0.4)	2.6 (0.3)	2.9 (0.4)	N/D	***0.0048***
** Benzyl alcohol**	149.6 (8.4)	201.0 (18.0)	**0.0007**	140.6 (9.4)	210.0 (14.4)	**<0.0001**	126.8 (4.0)	172.3740	154.5 (17.1)	247.5 (16.3)	0.0802
** Phenylethyl alcohol**	471.2 (77.9)	170.7 (40.0)	**<0.0001**	156.6 (20.9)	485 (80.2)	**<0.0001**	222.8 (7.5)	719.5645	90.3 (10.3)	251 (65.9)	**0.0005**
** 1-Dodecanol**	3.5 (0.3)	5.5 (0.7)	**0.0056**	5.1 (0.7)	4.0 (0.4)	0.0917	3.4 (0.3)	3.7 (0.4)	6.8 (1.0)	4.3 (0.7)	**0.0401**
** 3.3.6-Trimethyl-4.5-** ** heptadien-2-ol**	5.2 (1.6)	8.1 (2.8)	**0.0815**	N/D	13.3 (1.8)	**<0.0001**	N/D	10.4 (0.9)	N/D	16.2 (3.1)	0.0815
***Carbonyls***											
** 2-Butanone**	2.1 (0.6)	12.4 (1.8)	**<0.0001**	7.9 (1.3)	6.7 (2.6)	0.5234	4.2 (0.1)	N/D	11.5 (1.4)	13.3 (3.4)	0.1134
** 2-Methylbutanal**	3.4 (0.8)	11.2 (0.9)	**<0.0001**	7.6 (1.4)	6.9 (1.5)	0.6107	4.2 (1.4)	2.5 (0.8)	10.9 (1.4)	11.4 (1.3)	0.3809
** 3-Pentanone**	8.1 (0.6)	12.5 (0.7)	**<0.0001**	11.4 (1.0)	9.3 (0.8)	**0.0149**	8.6 (0.8)	7.6 (0.9)	14.2 (0.5)	10.9 (0.9)	0.1547
** Methyl butanoate**	5.7 (0.5)	8.8 (0.5)	**<0.0001**	8.6 (0.6)	59 (0.5)	**<0.0001**	7.1 (0.4)	4.3 (0.3)	10.2 (0.4)	7.5 (0.4)	0.9355
** Cyclohexanone**	1.8 (0.6)	1.8 (0.6)	0.9205	0.8 (0.4)	2.7 (0.6)	**0.0094**	N/D	3.643218	1.7 (0.6)	1.8 (1.2)	***0.0138***
** Acetoin**	6.1 (0.1)	47.8 (11.5)	**0.0025**	28.4 (10.6)	25.4 (9.9)	0.8047	6.3 (0.2)	5.9 (0.1)	50.6 (17.3)	45.0 (16.8)	0.8305
** 2.3.4.5-Tetramethyl-2-** ** cyclopentenone**	13.6 (4.1)	16.8 (4.0)	**0.0059**	1.9 (0.9)	28.5 (0.9)	**<0.0001**	N/D	27.2 (0.7)	3.8 (1.3)	29.8 (1.4)	0.6001
** Nonanal**	15.0 (1.6)	18.0 (2.1)	0.2626	16.8 (2.3)	16.2 (1.5)	0.8163	13.2 (1.7)	16.7 (2.7)	20.4 (3.9)	15.6 (1.4)	0.1320
** Decanal**	4.9 (0.2)	4.5 (0.3)	0.2685	4.8 (0.3)	4.6 (0.3)	0.7068	4.7 (0.2)	5.1 (0.4)	4.9 (0.6)	4.1 (0.3)	0.1201
** Benzaldehyde**	4.0 (1.3)	8.4 (3.1)	**0.0346**	12.5 (2.3)	N/D	**<0.0001**	8.1 (0.8)	N/D	16.9 (3.8)	N/D	***0.0346***
** Isophoron**	4.1 (0.2)	4.4 (0.3)	0.3670	3.8 (0.2)	4.8 (0.2)	**0.0022**	3.53 (0.2)	4.8 (0.2)	4.0 (0.4)	4.9 (0.3)	0.5649
** 3-Methylbenzaldehyde**	6.9 (1.5)	10.0 (2.1)	0.1354	4.4 (0.7)	12.5 (1.9)	**0.0006**	2.8 (0.3)	11.1 (1.7)	6.1 (1.0)	14.0 (3.5)	0.9280
** 2.4-Dimethylbenzaldehyde**	6.8 (2.2)	5.3 (2.1)	0.6480	5.3 (0.9)	6.8 (2.9)	0.6438	6.7 (1.6)	6.9 (4.4)	3.9 (0.6)	6.8 (4.3)	0.6763
***Esters***											
** Methyl acetate**	11.9 (2.6)	21.5 (3.3)	**<0.0001**	7.2 (1.1)	26.2 (2.0)	**<0.0001**	3.5 (0.2)	20.3 (1)	10.9 (0.4)	32.1 (1.4)	**0.0229**
** Ethyl acetate**	365.7 (105.4)	61.3 (5.0)	**<0.0001**	37.4 (6.2)	389.6 (98.4)	**<0.0001**	20.9 (1.0)	710.4 (37.3)	53.9 (7.9)	68.8 (5.1)	**<0.0001**
** Methyl isobutanoate**	1.8 (0.4)	2.8 (0.2)	0.0344	2.8 (0.4)	1.8 (0.3)	**0.0421**	2.4 (0.8)	1.2 (0.4)	3.2 (0.2)	2.4 (0.3)	0.6336
** Methyl** ** 2-methylbutanoate**	7.6 (0.6)	14.3 (0.6)	**<0.0001**	11.6 (1.0)	10.4 (1.3)	0.1406	8.9 (0.7)	6.4 (0.6)	14.3 (0.8)	14.3 (1.0)	0.1314
** Ethyl 2-methylbutanoate**	7.3 (2.3)	3.0 (0.9)	**<0.0001**	N/D	10.3 (1.5)		N/D	14.7 (1.6)	N/D	6.0 (0.4).	**<0.0001**
** (Z)-3-Hexenyl acetate**	8.7 (0.4)	16.5 (0.6)	**<0.0001**	12.9 (1.1)	12.3 (1.5)	0.4280	9.5 (0.5)	7.8 (0.6)	16.3 (0.7)	16.7 (1.1)	0.1802
** Ethyl lactate**	40.7 (12.3)	37.5 (10.4)	0.0792	1.6 (0.5)	76.6 (2.2)	**<0.0001**	N/D	81.5 (2.5)	3.3 (0.4)	71.7 (2.5)	***0.0015***
** Methyl salicylate**	4.5 (1.9)	2.2 (0.9)	0.1370	N/D	6.7 (1.6)		N/D	9.0 (2.7)	N/D	4.3 (1.4)	0.1370
** Ethyl hydrocinnamate**	3.3 (1.0)	1.5 (0.5)	**<0.0001**	N/D	4.8 (0.6)		N/D	6.6 (0.2)	N/D	3.0 (0.2)	***0.0000***
***Hydrocarbons***											
** 4-Methylnonane**	13.4 (1.5)	23.8 (1.6)	**<0.0001**	22.1 (2.1)	15.1 (1.8)	**0.0007**	16.6 (1.6)	10.2 (1.9)	27.5 (2.1)	20.0 (1.3)	0.7291
** 3-Methylnonane**	5.6 (1.7)	15.9 (0.9)	**<0.0001**	14.4 (1.2)	7.1 (2.1)	**<0.0001**	11.1 (1.0)	N/D	17.6 (1.2)	14.1 (0.8)	***0.0004***
** Decane**	41.0 (3.8)	70.1 (4.4)	**<0.0001**	62.7 (6.0)	48.4 (5.3)	**0.0127**	47.2 (3.8)	34.8 (5.9)	78.2 (6.8)	62.0 (3.8)	0.7167
** 2-Bornene**	55.0 (19.3)	91.1 (22.2)	0.0675	18.0 (7.9)	128.1 (17.7)	**<0.0001**	N/D	110.0 (20.8)	36.0 (12.1)	146.2 (28.5)	0.9959
***Others***											
** Dimethyl sulfide**	7.8 (0.7)	12.5 (1.0)	**0.0002**	9.6 (0.8)	10.7 (1.4)	0.3369	9.1 (0.9)	6.6 (1.0)	10.2 (1.3)	14.8 (0.9)	**0.0030**
** Methyl hexyl ether**	9.8 (1.4)	11.6 (0.9)	0.2767	11.6 (0.9)	9.8 (1.4)	0.2709	9.5 (0.9)	10.2 (2.8)	13.8 (0.7)	9.4 (1.0)	0.1246
** Theaspirane A**	50.1 (16.1)	45.0 (14.7)	0.7250	87.5 (13.6)	7.6 (1.00)	**<0.0001**	89.6 (22.8)	10.6 (0.5)	85.4 (17.2)	4.6 (0.7)	0.9524
** Theaspirane B**	59.2 (19.2)	55.8 (18.0)	0.8344	108.1 (15.2)	7.0 (0.7)	**<0.0001**	109.5 (24.7)	8.9 (0.5)	106.6 (20.0)	5.1 (0.8)	0.9767
** Coumaran**	47.7 (13.0)	3.8 (0.4)	**<0.0001**	46.9 (13.2)	4.7 (0.4)	**<0.0001**	90.1 (4.7)	5.4 (0.6)	3.7 (0.7)	4.0 (0.4)	**<0.0001**
***Phenols***											
** Creosol**	10.5 (3.2)	37.0 (10.0)	**<0.0001**	2.1 (1.5)	45.4 (7.4)	**<0.0001**	N/D	21.0 (1.0)	4.2 (2.7)	69.9 (2.1)	**<0.0001**
** 4-Ethylphenol**	1644.0 (95.8)	4.7 (0.5)	**<0.0001**	967.2 (291.3)	681.5 (206.7)	**<0.0001**	1931.2 (41.3)	1356.7 (75.0)	3.1 (0.4)	6.2 (0.4)	**<0.0001**
***Terpenes***											
**Linalool**	17.5 (0.5)	16.1 (0.6)	0.0971	16.8 (0.6)	16.8 (0.6)	0.9920	17.0 (0.6)	18.0 (0.7)	16.6 (1.0)	15.6 (0.7)	0.2348
** α-Terpineol**	13.2 (0.3)	11.8 (0.7)	**0.0494**	12.2 (0.8)	12.8 (0.3)	0.3658	13.9 (0.4)	12.6 (0.5)	10.5 (1.2)	13.1 (0.4)	**0.0118**
** ß-Damascenone**	8.1 (2.7)	11.2 (2.8)	0.2228	2.4 (0.9)	16.9 (2.2)	**<0.0001**	0.8 (0.5)	15.5 (3.1)	4.0 (1.4)	18.4 (3.4)	0.9637
** Isogeraniol**	2.5 (0.5)	3.2 (0.7)	0.3985	3.9 (0.2)	1.8 (0.8)	**0.0190**	3.5 (0.2)	1.5 (0.9)	4.3 (0.3)	2.1 (1.3)	0.9075
** Geraniol**	26 (0.1)	3.6 (0.2)	**<0.0001**	3.1 (0.2)	3.0 (0.2)	0.7207	2.5 (0.1)	2.6 (0.2)	3.7 (0.3)	3.5 (0.2)	0.5044

Notes: Means (standard error in parentheses). F-system, fermentation under interconnected, under-vacuum stainless-steel fermenters. G-system, fermentation in traditional independent fiberglass fermenters. N/D, not detected. N = number of independent values participating in the comparisons and contributing to means and standard errors.

**Table 3:** Volatile organic compounds (VOCs) specific to the F and G fermentation systems. Results of the ANOVA showing mean concentrations (µg/L) for fermentation system levels, fermentation periods, and their interaction, along with the corresponding *p*-values for each comparison.

Effect/Interaction	Fermentation System			Fermentation Period			FermentationSystem×Fermentation Period				
Factor Level	F	G	*p*-Value	9 Days	55 Days	*p*-Value	F	F	G	G	*p*-Value
							9 Days	55 Days	9 Days	55 Days	
N	12	12		12	12		6	6	6	6	
VOCs exclusively detected in the interconnected–under–vacuum–stainless-steel fermenters (F-system)											
***Acids***											
** Butanoic acid**	5.1 (1.6)	N/D		N/D	5.1 (1.6)		N/D	10.3 (0.5)	N/D	N/D	
***Alcohols***											
** 2-Ethylbutanol**	3.2 (1.0)	N/D		N/D	3.2 (1.0)		N/D	6.4 (0.4)	N/D	N/D	
** 3-Methylpentanol**	3.9 (0.4)	N/D		1.3 (0.4)	2.6 (0.8)	**<0.0001**	2.7 (0.2)	5.2 (0.2)	N/D	N/D	**<0.0001**
** 1-Heptanol**	3.6 (1.1)	N/D		3.6 (1.1)	N/D		7.2 (0.2)	N/D	N/D	N/D	
** 6-Methyl-5-hepten-2-ol**	2.6 (0.8)	N/D		2.6 (0.8)	N/D		5.2 (0.6)	N/D	N/D	N/D	
***Carbonyls***											
** 2.3-Pentanedione**	2.0 (0.6)	N/D		2.0 (0.6)	N/D		4.0 (0.3)	N/D	N/D	N/D	
** Octanal**	2.6 (0.7)	N/D		0.7 (0.5)	1.9 (0.7)	0.0792	1.3 (0.9)	3.8 (1.0)	N/D	N/D	0.0792
***Esters***											
** Ethyl propanoate**	2.8 (0.9)	N/D		N/D	2.8 (0.9)		N/D	5.7 (0.4)	N/D	N/D	
** n-Propyl acetate**	3.1 (1.0)	N/D		N/D	3.1 (1.0)		N/D	6.3 (0.3)	N/D	N/D	
** Isobutyl acetate**	3.0 (0.9)	N/D		N/D	3.0 (0.9)		N/D	5.9 (0.5)	N/D	N/D	
** Ethyl 3-methylbutanoate**	3.9 (1.2)	N/D		N/D	3.9 (1.2)		N/D	7.8 (0.8)	N/D	N/D	
** Isopentyl acetate**	9.4 (2.2)	N/D		1.2 (0.4)	8.2 (2.5)	**<0.0001**	2.5 (0.2)	16.3 (1.3)	N/D	N/D	**<0.0001**
** Methyl hexanoate**	1.1 (0.5)	N/D		1.1 (0.5)	N/D		2.3 (0.8)	N/D	N/D	N/D	
** Ethyl hexanoate**	3.0 (0.9)	N/D		N/D	3.0 (0.9)		N/D	5.9 (0.6)	N/D	N/D	
** Ethyl 3-hydroxybutanoate**	1.7 (0.6)	N/D		N/D	1.7 (0.6)		N/D	3.4 (0.5)	N/D	N/D	
** Methyl 4-methylaminobenzoate**	3.4 (1.1)	N/D		N/D	3.4 (1.1)		N/D	6.7 (0.6)	N/D	N/D	
**VOCs exclusively detected in the traditional independent fiberglass fermenters (G-system)**											
***Acids***											
** Propanoic acid**	N/D	5.0 (0.5)		2 (0.6)	3.1 (1.0)	**0.0108**	N/D	N/D	3.9 (0.5)	6.1 (0.6)	**0.0107**
** Nonanoic acid**	N/D	7.8 (1.4)		3.1 (1.3)	4.7 (1.7)	0.2757	N/D	N/D	6.3 (1.9)	9.3 (2.0)	**0.2757**
***Carbonyls***											
** 3-Methylbutanal**	N/D	3.6 (0.4)		1.8 (0.6)	1.8 (0.6)	0.9046	N/D	N/D	3.7 (0.6)	3.6 (0.6)	0.9046
** 4-Methyl-3-penten-2-one**	N/D	8.1 (1.9)		1.5 (0.5)	6.6 (2.2)	**0.0002**	N/D	N/D	3.0 (0.3)	13.1 (2.2)	**0.0002**
** 2-Heptanone**	N/D	3.3 (0.6)		2.3 (0.7)	0.9 (0.4)	**0.0015**	N/D	N/D	4.7 (0.5)	1.9 (0.6)	**0.0015**
** 6-Methyl-5-hepten-2-one**	N/D	3.1 (0.2)		1.60.5)	1.5 (0.5)	0.3765	N/D	N/D	3.2 (0.1)	2.9 (0.3)	0.3765
** Acetophenone**	N/D	1.4 (0.5)		N/D	1.4 (0.5)		N/D	N/D	N/D	2.9 (0.4)	
***Esters***											
** Methyl lactate**	N/D	14.6 (4.4)		N/D	14.6 (4.4)		N/D	N/D	N/D	29.2 (0.9)	
** Benzyl acetate**	N/D	2.8 (0.1)		1.3 (0.4)	1.5 (0.5)	**0.0364**	N/D	N/D	2.5 (0.2)	3.0 (0.1)	0.0364
** Methyl hydrocinnamate**	N/D	4.2 (0.5)		1.7 (0.6)	2.5 (0.8)	**0.0411**	N/D	N/D	3.4 (0.6)	5.1 (0.5)	**0.0411**
***Hydrocarbons***											
** 2.5-Dimethylnonane**	N/D	2.7 (0.1)		1.5 (0.5)	1.2 (0.4)	**0.0020**	N/D	N/D	3.0 (0.1)	2.4 (0.1)	**0.0020**
***Phenols***											
** o-Guaiacol**	N/D	76.0 (32.4)		36.5 (24.6)	39.5 (26.6)	0.9320	N/D	N/D	73.0 (46.2)	78.9 (49.9)	0.9320
** Phenol**	N/D	6.3 (2.7)		3.1 (2.1)	3.3 (2.2)	0.9367	N/D	N/D	6.1 (3.9)	6.6 (4.2)	0.9367
***Terpenes***											
** Dihydroedulan II**	N/D	5.5 (1.8)		3.1 (1.4)	2.4 (1.6)	0.7279	N/D	N/D	6.1 (2.1)	4.8 (3.1)	0.7279

Notes: Standard error in parentheses. Significant *p*-values in bold. F-system, fermentation in an interconnected system, under vacuum stainless-steel fermenters. G-system, fermentation in traditional independent fiberglass fermenters. N/D, not detected. N = number of independent values participating in the comparisons and contributing to means and standard errors.

### 3.5. Relationship Between Microbial Population and VOC Profile

Although numerous studies have described Spanish-style volatile profiles under various conditions, fewer have identified the microbial or enzymatic origins of these VOCs. The present study aims to tentatively associate the relative abundances (sequencing data) of bacterial and fungal genera identified on day 22 in both fermentation systems (Table S4) with their VOC profiles using Partial Least Squares Regression (PLS-R) [14]. Analyses were conducted separately for common versus system–specific taxa and VOCs.

**Figure 4:**
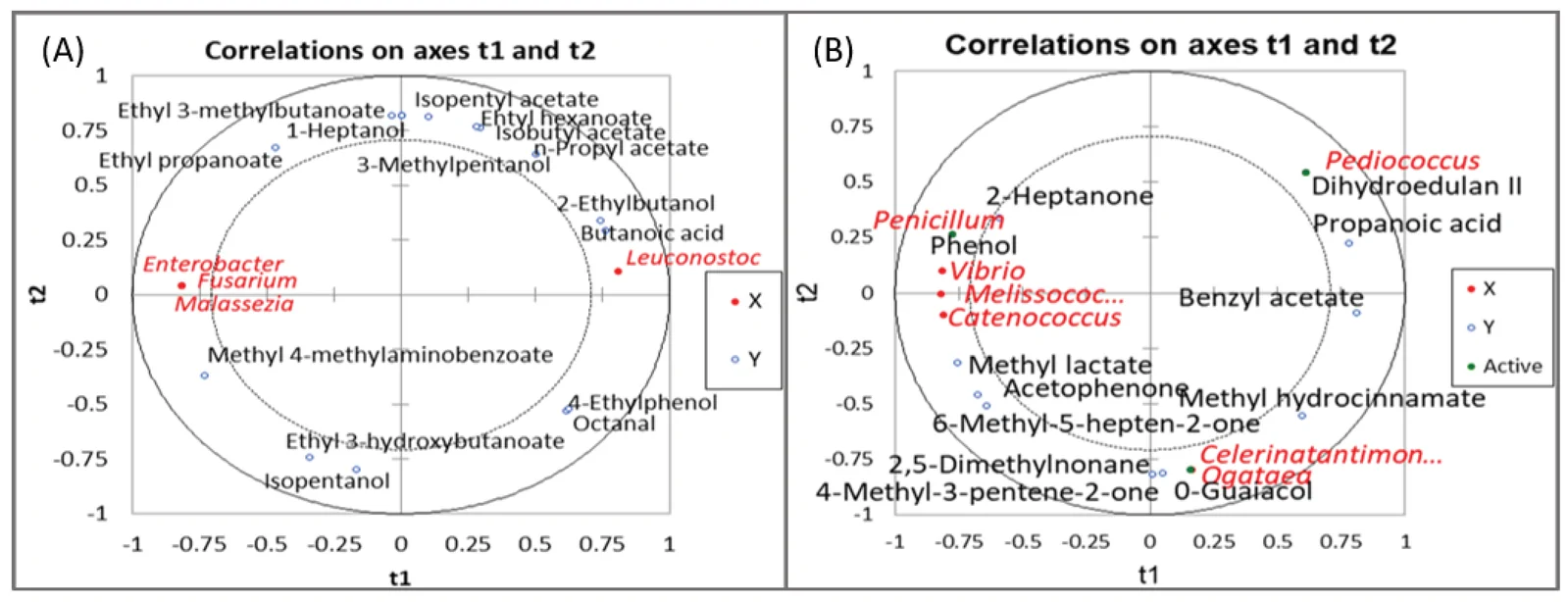
Projection of taxa and VOCs onto the t1-t2 plane after PLS-R analysis. Relationship between exclusive taxa and VOCs in (**A**) the interconnected stainless-steel system (F) and (**B**) the traditional independent system (G). X represents microbial genera; Y represents volatile compounds.

*F-system.* The PLS–R model for the F-system showed strong representation of both the independent (R^2^X = 0.667) and dependent (R^2^Y = 0.667) variables, with excellent predictive ability (Q^2^ = 1.00). All projections onto t1 and t2 fell within the 0.95 confidence ellipse (Figure 4A). The score plot clearly separated *Leuconostoc* (right) from *Enterobacter*, *Fusarium*, and *Malassezia* (left). Correspondingly, VOCs on the right may be related to *Leuconostoc*, whereas those on the left may be associated with the latter three genera. Standardized coefficients (regression coefficients scaled to enable comparison of the relative contribution of each predictor variable) showed associations between *Leuconostoc* and butanoic acid (2.331), 2-ethylbutanol (2.648), 3-methylpentanol (4.721), n-propyl acetate (5.508), isobutyl acetate (5.545), isopentyl acetate (5.801), and hexyl hexanoate (5.817). *Enterobacter*, *Fusarium*, and *Malassezia* showed moderate associations with 1-heptanol (1.923), ethyl propanoate (1.723), ethyl 3-methylbutanoate (1.933), and methyl 4-methylaminobenzoate (0.870).

*G-system*. The PLS-R model for the traditional G-system also performed well (R^2^X = 0.667; R^2^Y = 0.650; Q^2^ = 0.945. All taxa–volatile projections fell within the 0.95 confidence ellipse (Figure 4). *Pediococcus* was positively associated with Dim 1, whereas *Penicillium*, *Vibrio*, *Melissococcus*, and *Catenococcus* were negatively associated. *Celerinatantimonas* and *Ogataea* correlated with Dim 2, though their low VIP scores (0.153 and 0.161, respectively) suggest they may load on other dimensions. VOCs on the right of the plot corresponded to *Pediococcus*, those on the left to *Penicillium*, *Vibrio*, *Melissococcus*, and *Catenococcus*, and those at the bottom to *Celerinatantimonas* and *Ogataea*. Standardized coefficients confirmed that *Pediococcus* was associated with propanoic acid (0.242), benzyl acetate (0.145), and dihydroedulan II (0.318). The negatively associated microbial genera contributed similarly (coefficient ranges in parentheses) to 2-heptanone (0.140–0.176), 6-methyl-5-hepten-2-one (0.152–0.222), acetophenone (0.106–0.221), methyl lactate (0.146–2.31), and phenol (0.190–0.231). *Celerinatantimonas* and *Ogataea* were mainly linked to methyl hydrocinnamate (0.287, 0.288), 2,5-dimethylnonane (0.393, 0.392), and o-guaiacol (0.390, 0.390). *Pediococcus* is frequently reported in both naturally fermented table olives and Spanish-style [27]. This genus is broadly found in natural and traditional fermentations in Italy, Greece, Spain, and Portugal [32].

**Figure 5:**
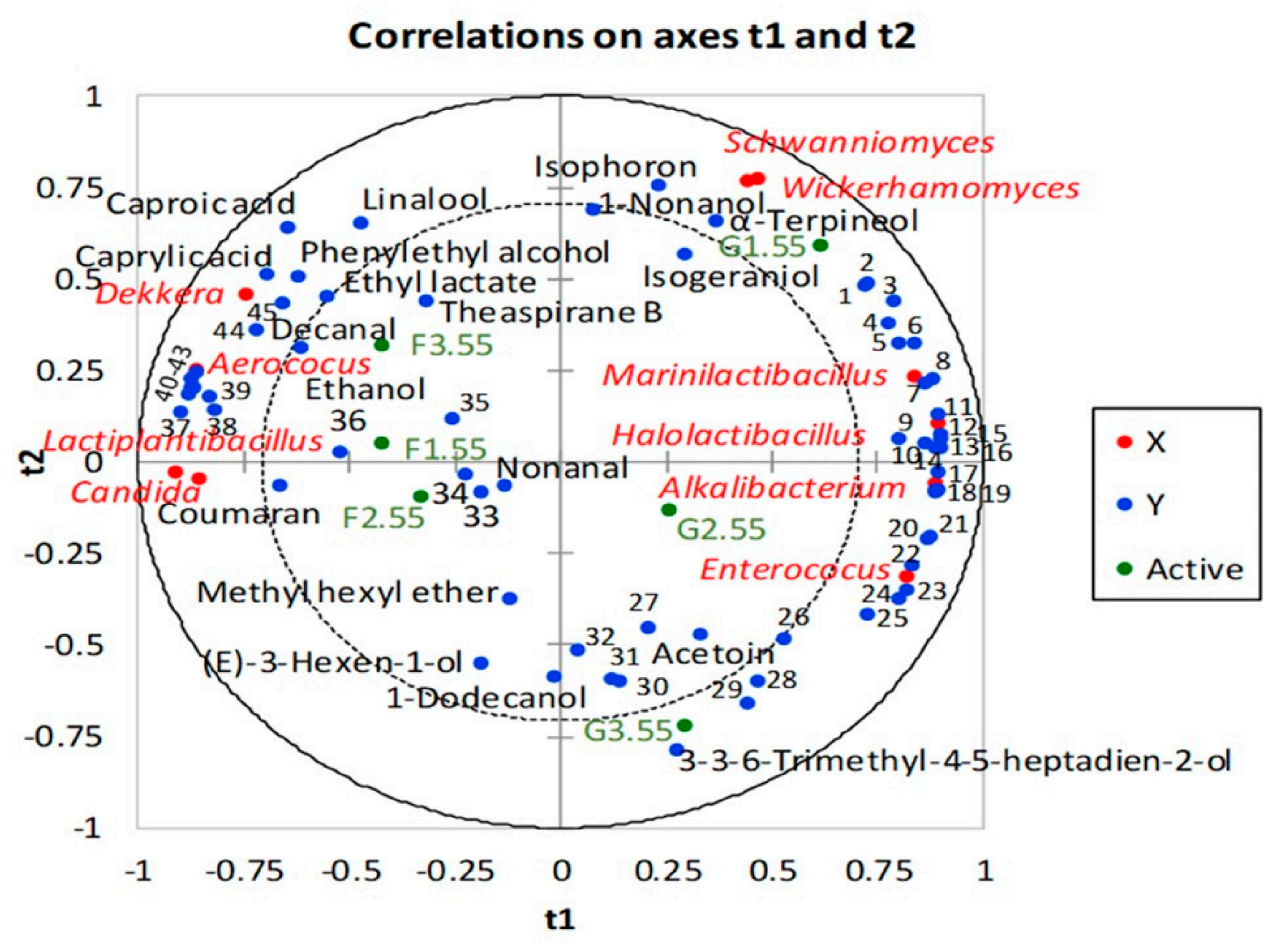
Projection of taxa and VOCs onto the t1-t2 plane after PLS-R analysis. Relationship between common taxa and VOCs in the interconnected stainless-steel (F-system) and traditional independent fermenter systems (G-system). Numbers correspond to the following compounds: **1**, 3-methyl-3-buten-1-ol; **2**, 2-pentanol; **3**, **2**-butanol; **4**, geraniol; **5**, 1-octanol; **6**, benzyl alcohol; **7**, prenol; **8**, 1-hexanol; **9**, (Z)-3-hexen-1-ol; **10**, (Z)-3-hexenyl acetate; **11**, acetic acid; **12**, methylacetate; **13**, 5-hexen-1-ol; **14**, 4-penten-1-ol; **15**, 1-pentanol; **16**, 6-hepten-1-ol; **17**, creosol; **18**, decane; **19**, 3-pentanol; **20**, 4-methylnonane; **21**, 3-methylnonane; **22**, dimethyl sulfide; **23**, methyl 2-methylbutanoate; **24**, methyl butanoate; **25**, 2-methylbutanal; **26**, 2-butanone; **27**, 2,3,4,5-tetramethyl-2-cyclopentanone; **28**, 3-methylbenzaldehyde; **29**, β-damascenone; **30**, methyl isobutanoate; **31**, 3-pentanone; **32**, 2-Bbrnene; **33**, 3-cyclopentyl-1-propanol; **34**, 2,4-dimethylbenzaldehyde; **35**, methyl salicylate; **36**, cyclohexanone; **37**, ethyl hydrocinnamate; **38**, ethyl 2-methylbutanoate; **39**, 2-ethylhexanol; **40**, 1-propanol; **41**, isobutanol; **42**, ethyl acetate; **43**, 1-decanol; **44**, 2-methylbutanoic acid; **45**, theaspirane A.

*Common microorganisms and typical VOCs*. Associating common taxa with VOC characteristics may be more reliable because they have larger, more representative populations. For this purpose, sequences from common bacterial and fungal taxa (Table S2) were linked to typical VOC concentrations on day 55. The four-component PLS-R model explained substantial variance in both the independent (R^2^X = 0.829) and dependent (R^2^Y = 0.772) variables. Overall predictive ability (Q^2^ = 0.584) was moderate (R^2^ > 0.90; low RMSE; Table S3), but high for most individual VOCs. Figure 5 provides an intuitive overview of microbial-volatile relationships. Only selected volatiles are labeled to reduce clutter. Projections of the fermenters show clear segregation: G-system fermenters on the right, and F-system on the left. Dim1 reflects the processing system, while Dim2 lacks a clear experimental interpretation. F-system fermenters were more uniform, whereas the G-system exhibited greater dispersion, indicating higher microbial and VOC variability. *Aerococcus*, *Lactiplantibacillus*, and *Candida* were associated with coumaran and compounds 36–43 (cyclohexanone to 1-decanol). At the same time, *Dekkera* correlated to decanal, ethyl lactate, phenylethyl alcohol, caproic acid, caprylic acid, or compounds 44 (2-methylbutanoic acid) and 45 (theaspirane A). Conversely, *Schwanniomyces*, *Wickerhamomyces*, *Marinilactibacillus*, *Halolactibacillus*, *Alkalibacterium*, *and Enterococcus* were associated with the G-system and VOCs on the right (Figure 5).

Thus, *Schwanniomyces* and *Wickerhamomyces* correlated with α-terpineol, isogeraniol, or isophoron, as well as with compounds 1–4 (i.e., from 3-methyl-3-buten-1-ol to geraniol). *Marinilictibacillus*, *Halolactibacillus*, *Alkalibacterium*, and *Enterococcus* were linked to compounds 5–25 (ranging from 1-octanol to 2-methylbutanal). Volatile compounds located near the origin (e.g., nonanal) are poorly represented and likely contribute to another model dimension.

*Microbial contributions derived from standardized coefficients*. The contributions of microorganisms to specific VOCs were also quantified using the significant standardized coefficients from their respective models (Table S3). These coefficients indicate that *Halolactibacillus* and *Wickerhamomyces* predominantly contributed to acetic acid, with minor input from *Schwanniomyces*, as reflected in their model coefficients. Ethanol production is driven by *Dekkera* and *Candida*, with additional contributions from *Lactiplantibacillus and Aerococcus*, and limited participation from *Wickerhamomyces and Schwanniomyces*. Many alcohols show strong microbial associations. In contrast, acetoin is associated exclusively with *Halolactibacillus.* Most VOCs exhibited associations with two genera, with additional minor links to others, making interpretation complex. The detailed information on significant associations (standardized coefficients) is provided in Table S3.

**Figure 6:**
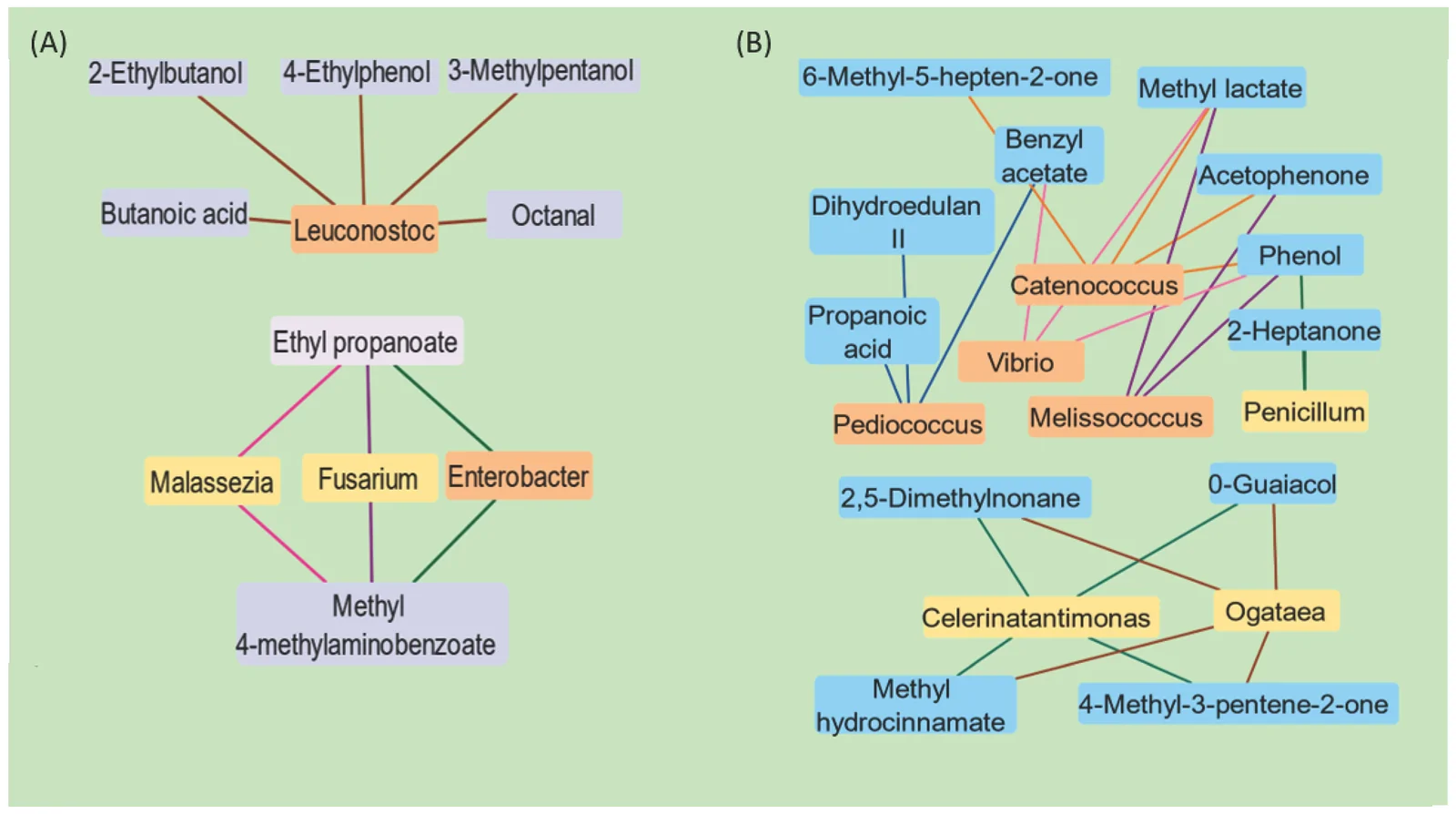
Visualization of significant correlations between specific volatile compounds and taxa in the F-system (**A**) (interconnected fermentation) and the G-system (**B**) (traditional independent processing). Nodes represent volatile compounds (purple, blue and pink), bacteria (orange), and fungi (yellow).

*Correlation-based visualization.* Multiple associations of VOCs with microbial populations, as indicated by significant correlations, can also be visualized graphically. F-system (Figure 6A) showed few associations. *Leuconostoc* was linked to butanoic acid, 2-ethylbutanol, 4-ethylphenol, 3-methylpentanol, and octanal. *Enterobacter* (bacteria) or *Malassezia* and *Fusarium* (fungi) were associated with ethyl propanoate and methyl 4-methylaminobenzoate. G-system (Figure 6B) exhibited several associations, with bacteria contributing to more VOCs than fungi. Most taxa were linked to several compounds, as indicated by the connecting lines. Only a few volatiles (e.g., 2,5-dimethylnonane, o-guaiacol, methyl hydrocinnamate, 4-methyl-3-pentene-2-one) were associated with exactly two genera (*Celerinatantimonas* and *Ogatea*). Methyl lactate and phenol correlated with multiple taxa. Further associations are shown directly in Figure 6.

**Figure 7:**
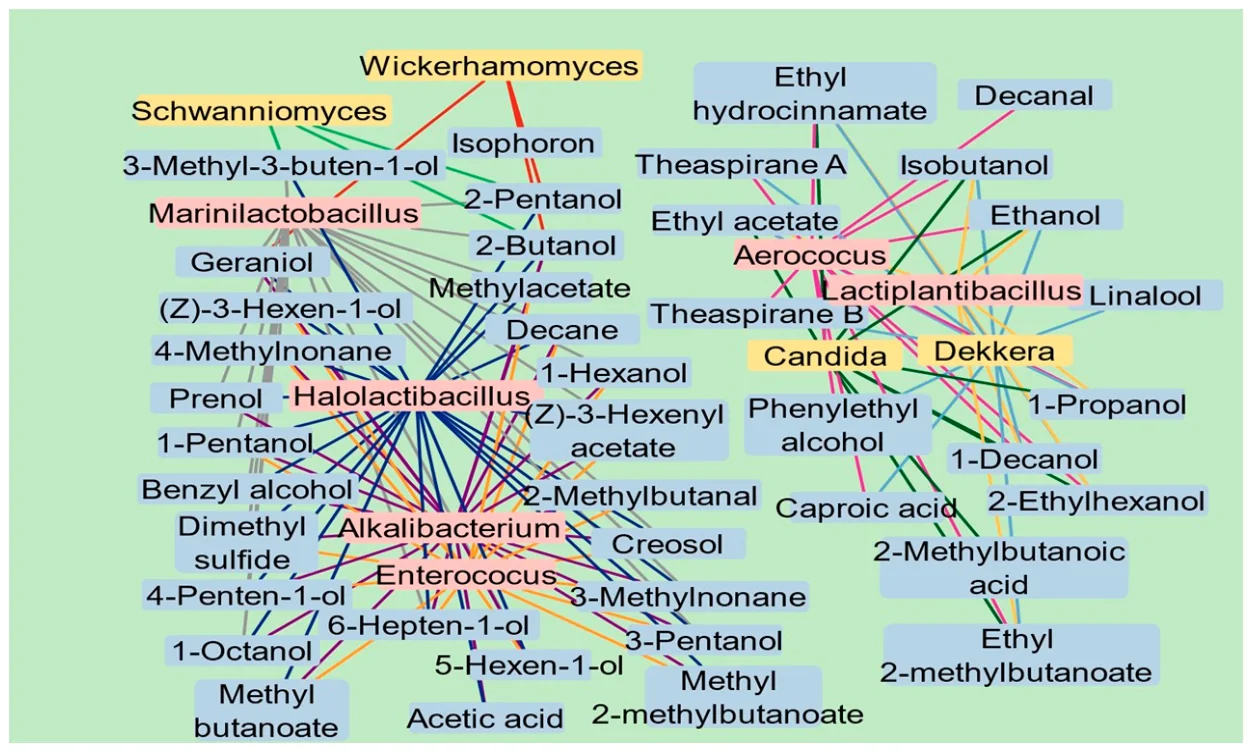
Visualization of significant correlations between common volatile compounds and taxa in the F-system (interconnected fermentation) and the G-system (traditional independent processing). Nodes represent volatile compounds (blue), bacteria (pink), and fungi (yellow).

*Common VOCs across systems.* The association between common VOCs and microbial taxa was complex given the large number of VOCs and taxa. As shown in Figure 7, *Halolactibacillus* and *Marinilactibacillus* were significantly associated with most relevant VOCs, followed by *Alkalibacterium*, *Enterococcus*, and *Aerococcus. Lactiplantibacillus* contributed to only seven major volatiles (ethyl hydrocinnamate, isobutanol, ethanol, 1-propanol, 2-ethylhexanol, 1-decanol, and ethyl 2-methylbutanoate). Among fungi, *Dekkera* and *Candida* were the primary contributors to several VOCs, whereas *Schwanniomyces*—linked to 2-pentanol, 2-butanol, and 3-methyl-3-buten-1-ol—and *Wickerhamomyces*—related to geraniol, 2-pentanol, and 2-butanol—were associated with only three VOCs. The relatively limited contribution of *Lactiplantibacillus* to key VOCs—and thus the sensory profile of Spanish-style olives—may explain the poor acceptance of starter cultures composed solely of this genus [26].

The findings of this study are consistent with previous reports linking microbial populations and starter cultures to VOC production in table olives, which demonstrated species-dependent VOC profiles among yeasts, with several compounds shared with naturally fermented olives [20,25]. Similarly, specific VOC biomarkers have been associated with different LAB–yeast starter combinations [19]. Spoilage-associated VOCs have also been linked to particular taxa, including *Propionibacterium* and *Ruminococcus*, responsible for “zapatera” and “butyric” defects, respectively [33] Moreover, the value of integrating amplicon metagenomics to unravel microbe–VOC relationships and improve understanding of table olive fermentations was highlighted [30].

Overall, this study presents the primary comparative assessment of volatile profiles and microbial populations between a novel vacuum fermentation system and traditional processes. Although the experimental design does not elucidate the precise regulatory mechanism—which would require evaluating additional variables such as dissolved oxygen, redox potential, and gas composition—the empirical evidence demonstrated that vacuum operation and vessel configuration influence fermentation dynamics.

## 4. Conclusions

This study demonstrated that a novel vacuum-operated system of interconnected fermenters substantially alters the VOC profile of Spanish-style table olives compared with the traditional independent fermentation process. Although a limited set of VOCs characterized each system, most compounds were shared between processes, indicating a common fermentation basis despite their technological differences. Alcohols, acids, esters, and carbonyl compounds were the dominant VOC groups and generally increased throughout fermentation. Only four VOCs—decanal, 2,4-dimethylbenzaldehyde, methyl ethyl ether, and linalool—remained unaffected by the fermentation system or time, suggesting they originated during lye treatment or the early stages of processing.

Most VOCs differed significantly in concentration across systems. The interconnected under-vacuum system (F) was associated with a more limited set of compounds, including isobutanol, caproic acid, ethyl 2-methylbutanoate, and ethyl hydrocinnamate. In contrast, the traditional system (G) was linked to a broader range of VOCs, including 3-pentanol, (Z)-3-hexenyl acetate, (Z)-3-hexen-1-ol, and prenol. Overall, the traditional G-system produced a more complex volatile profile, whereas the F-system generated a more streamlined and potentially more controllable profile.

Microbial–VOC analyses consistently differentiated microbial communities across the fermentation system. The F-system was primarily associated with typical Spanish-style fermentation genera, including *Lactiplantibacillus*, *Candida*, *Dekkera*, and *Leuconostoc*, whereas the G-system harbored a more diverse microbial community linked to a broader range of VOCs. These associations were supported by PLS-R models, standardized coefficients, and correlation networks, reinforcing their biological relevance and their potential value as system-specific biomarkers.

Linking specific microbial populations to VOC formation offers new opportunities to design starter cultures and microbial consortia that modulate volatile profiles and enhance product consistency. Overall, the interconnected under-vacuum fermentation system represents a promising technological alternative for producing Spanish-style table olives with more homogeneous fermentation performance and greater process controllability.

## Data Availability

The original contributions presented in the study are included in the article/Supplementary Material, further inquiries can be directed to the corresponding author.

## Supplementary Materials

The following supporting information can be downloaded at: https://www.mdpi.com/article/10.3390/foods15173034/s1. Figure S1. Mean and standard deviations of common VOCs in the Spanish-style Manzanilla cultivar, processed in interconnected under-vacuum fermenters (F-system) and traditional independent fermenters (G-system). The graph illustrates patterns for each fermenter within each process across the two fermentation periods. Values were obtained from ANOVA analysis. Compounds are numbered in parentheses for reference in the text; Figure S2. Mean values and confidence limits of VOCs in the Spanish-style Manzanilla cultivar, showing significant interactions between fermentation systems (F and G) and fermentation periods. The graph illustrates overall patterns for each system and fermentation period. Values were obtained from ANOVA analysis. Compounds are numbered in brackets for reference in the text; Table S1. Volatile organic compounds (VOCs) detected in the F and G green Spanish-style table olive fermentation systems. The table shows the chemical class, compound name, CAS number, identification method (MS, RI, Std), and relative concentration (µg/L, referenced to 6-chloro-2-hexanone) at 9 and 55 days. The F-system uses interconnected, under-vacuum stainless-steel fermenters, and the G-system uses traditional independent fiberglass fermenters; Table S2. Study of the volatile organic compounds (VOCs) detected in the F and G fermentation systems. Taxa sequences and bacterial and fungal genera identified in the F- and G-systems; Table S3. Volatile organic compounds (VOCs) detected in the F and G fermentation systems. Contribution of each identified taxon to predicting common volatile concentrations, based on the standardized PLS regression coefficients; Table S4. Study of VOCs detected in the F and G fermentation systems. Pearson correlation matrix between the identified microbial taxa and VOC levels in the F-system; Table S5. Study of VOCs detected in the F and G fermentation systems. Pearson correlation matrix between the identified taxa and VOC levels in the G-system.
