# Supplementary material for: Volatile Dynamics and Their Associations with Microbial Genera in Spanish-Style Table Olives Processed in Interconnected Under-Vacuum and Fiberglass Independent Vessels
Source: Foods. 2026 Aug 27;15(17):3034. doi: 10.3390/foods15173034 (PMC13565360; doi:10.3390/foods15173034)
Supplement: Supplementary file 1 [file foods-15-03034-s001.zip › Figure S1.pdf]

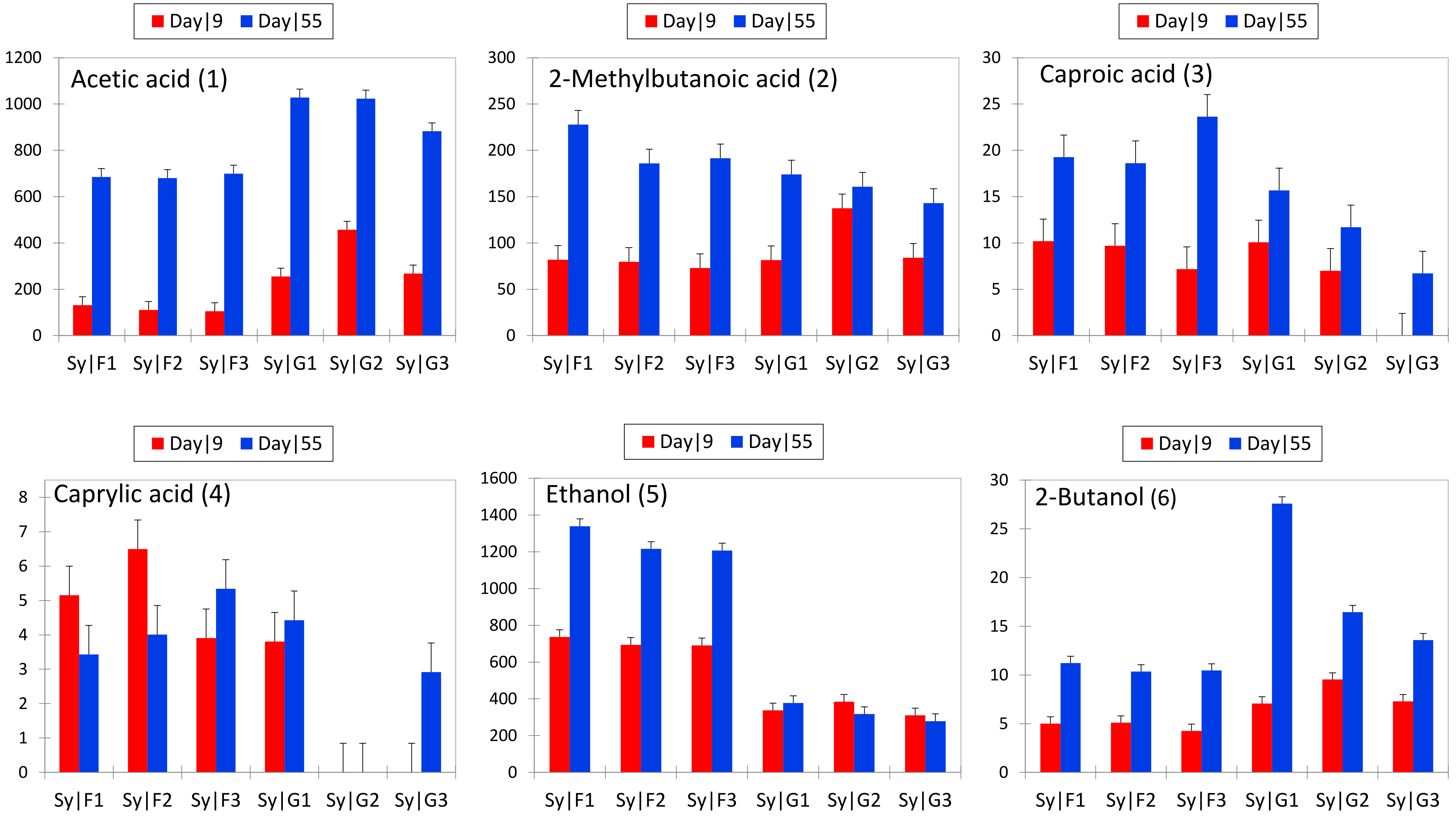

Day|9
Day|55
Acetic acid (1)
2-Methylbutanoic acid (2)
Caproic acid (3)
Caprylic acid (4)
Ethanol (5)
2-Butanol (6)
Sy|F1
Sy|F2
Sy|F3
Sy|G1
Sy|G2
Sy|G3

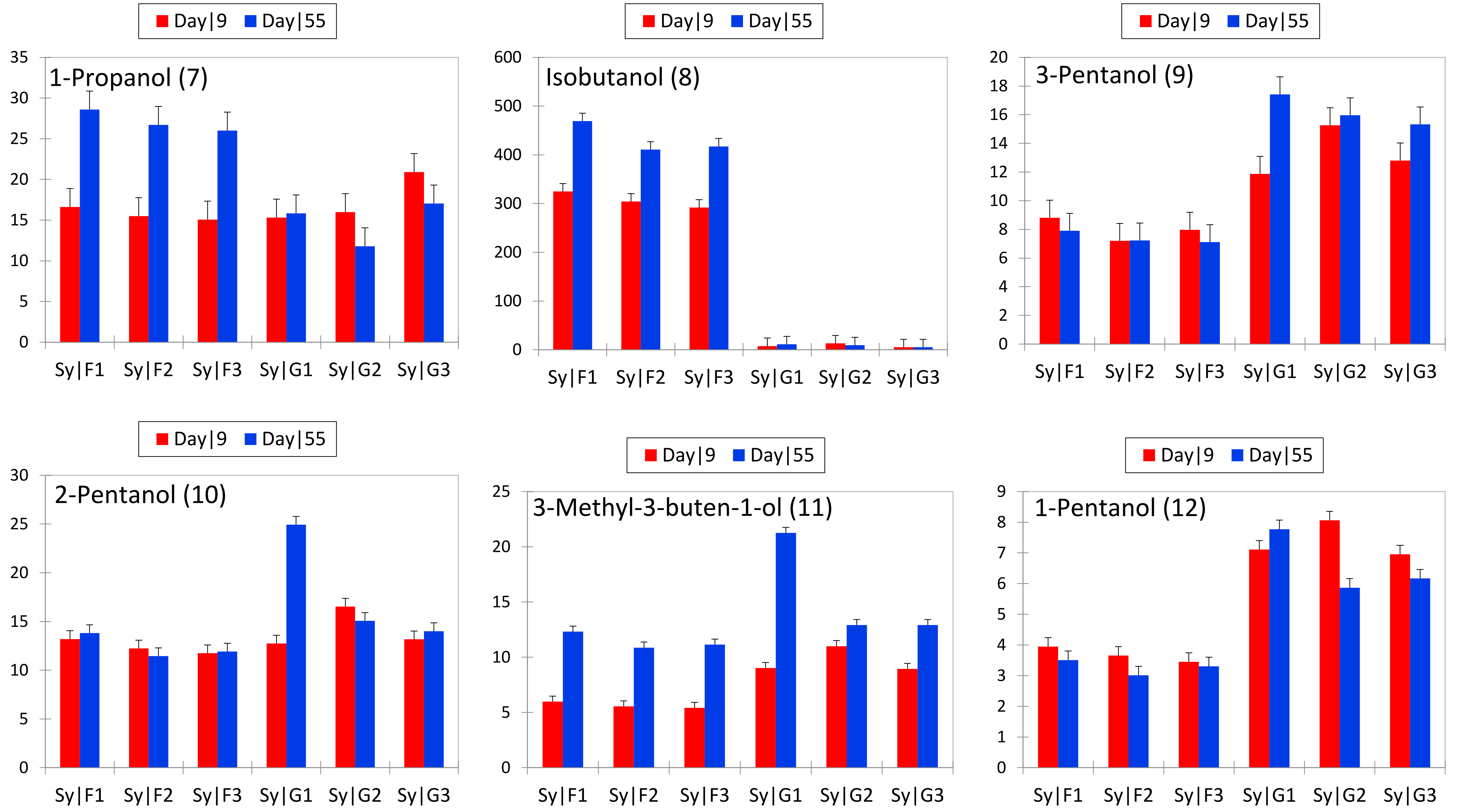

Day|9 Day|55
1-Propanol (7)
0 5 10 15 20 25 30 35
Sy|F1 Sy|F2 Sy|F3 Sy|G1 Sy|G2 Sy|G3
Isobutanol (8)
0 100 200 300 400 500 600
3-Pentanol (9)
0 2 4 6 8 10 12 14 16 18 20
2-Pentanol (10)
0 5 10 15 20 25 30
3-Methyl-3-buten-1-ol (11)
0 5 10 15 20 25
1-Pentanol (12)
0 1 2 3 4 5 6 7 8 9

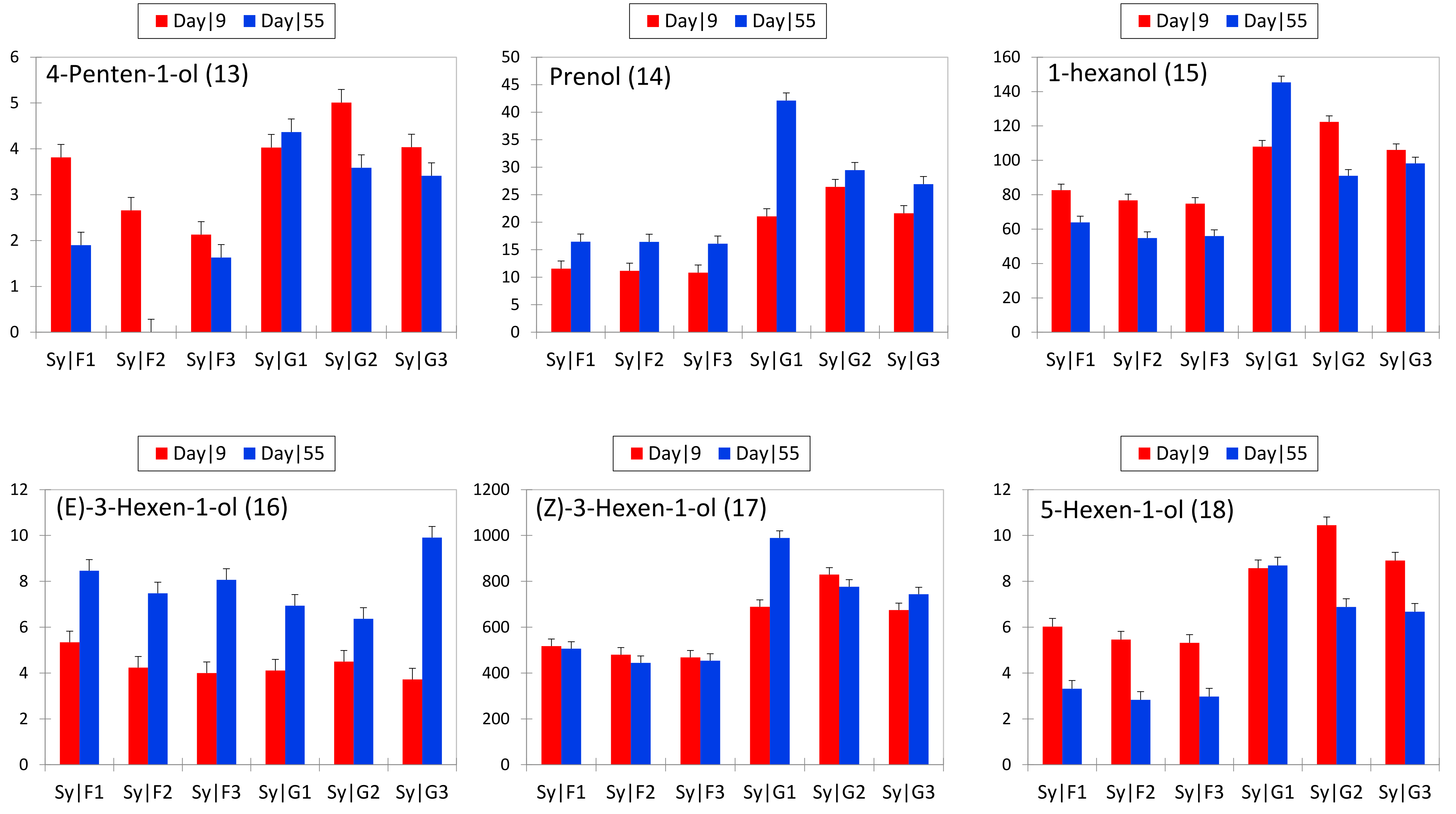
Day|9 Day|55
4-Penten-1-ol (13)
0 1 2 3 4 5 6
Sy|F1 Sy|F2 Sy|F3 Sy|G1 Sy|G2 Sy|G3
Day|9 Day|55
Prenol (14)
0 5 10 15 20 25 30 35 40 45 50
Sy|F1 Sy|F2 Sy|F3 Sy|G1 Sy|G2 Sy|G3
Day|9 Day|55
1-hexanol (15)
0 20 40 60 80 100 120 140 160
Sy|F1 Sy|F2 Sy|F3 Sy|G1 Sy|G2 Sy|G3
Day|9 Day|55
(E)-3-Hexen-1-ol (16)
0 2 4 6 8 10 12
Sy|F1 Sy|F2 Sy|F3 Sy|G1 Sy|G2 Sy|G3
Day|9 Day|55
(Z)-3-Hexen-1-ol (17)
0 200 400 600 800 1000 1200
Sy|F1 Sy|F2 Sy|F3 Sy|G1 Sy|G2 Sy|G3
Day|9 Day|55
5-Hexen-1-ol (18)
0 2 4 6 8 10 12
Sy|F1 Sy|F2 Sy|F3 Sy|G1 Sy|G2 Sy|G3

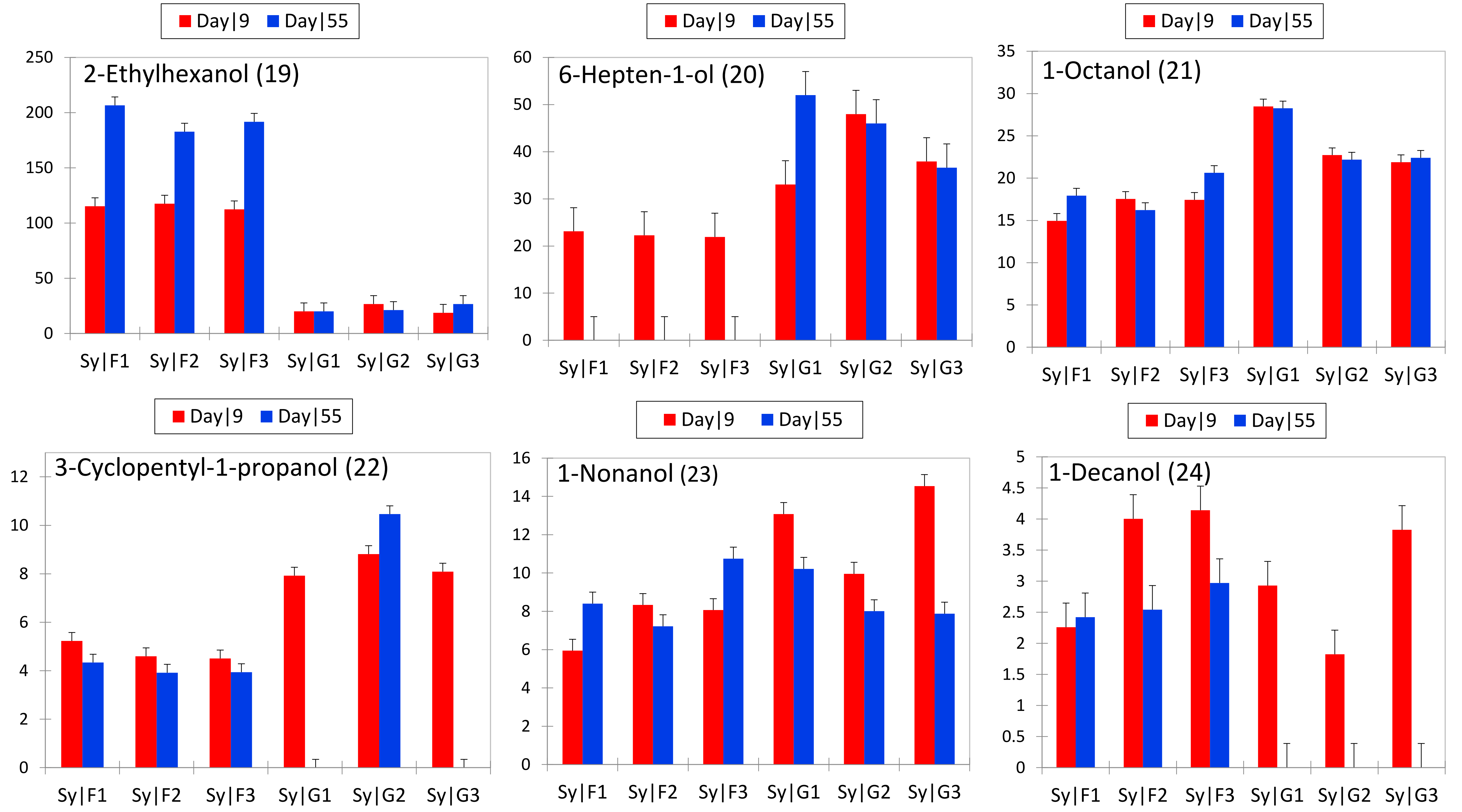

Day|9
Day|55
2-Ethylhexanol (19)
250
200
150
100
50
0
Sy|F1
Sy|F2
Sy|F3
Sy|G1
Sy|G2
Sy|G3
Day|9
Day|55
6-Hepten-1-ol (20)
60
50
40
30
20
10
0
Sy|F1
Sy|F2
Sy|F3
Sy|G1
Sy|G2
Sy|G3
Day|9
Day|55
1-Octanol (21)
35
30
25
20
15
10
5
0
Sy|F1
Sy|F2
Sy|F3
Sy|G1
Sy|G2
Sy|G3
Day|9
Day|55
3-Cyclopentyl-1-propanol (22)
12
10
8
6
4
2
0
Sy|F1
Sy|F2
Sy|F3
Sy|G1
Sy|G2
Sy|G3
Day|9
Day|55
1-Nonanol (23)
16
14
12
10
8
6
4
2
0
Sy|F1
Sy|F2
Sy|F3
Sy|G1
Sy|G2
Sy|G3
Day|9
Day|55
1-Decanol (24)
5
4.5
4
3.5
3
2.5
2
1.5
1
0.5
0
Sy|F1
Sy|F2
Sy|F3
Sy|G1
Sy|G2
Sy|G3

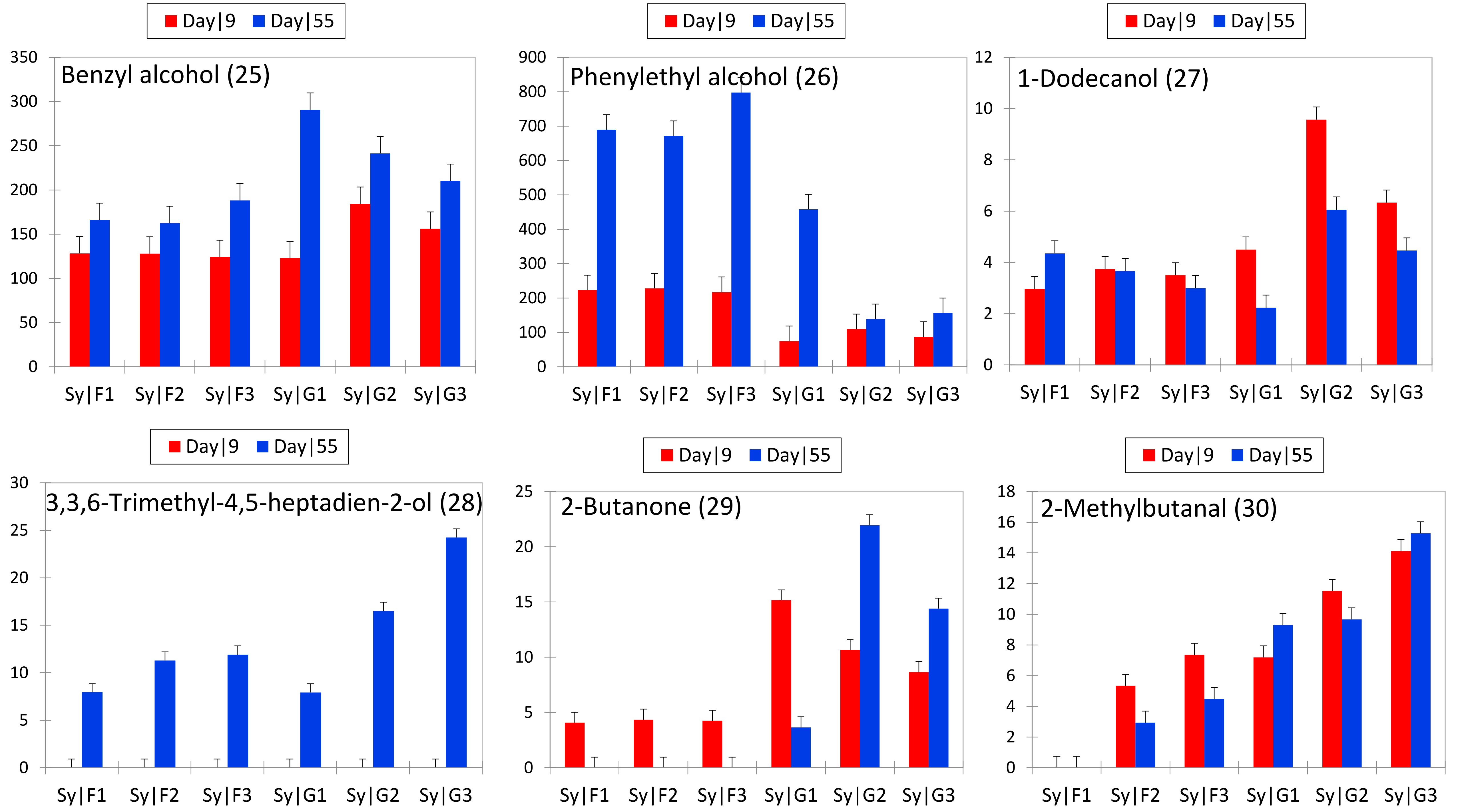
Day|9
Day|55
Benzyl alcohol (25)
Phenylethyl alcohol (26)
1-Dodecanol (27)
3,3,6-Trimethyl-4,5-heptadien-2-ol (28)
2-Butanone (29)
2-Methylbutanal (30)
Sy|F1
Sy|F2
Sy|F3
Sy|G1
Sy|G2
Sy|G3

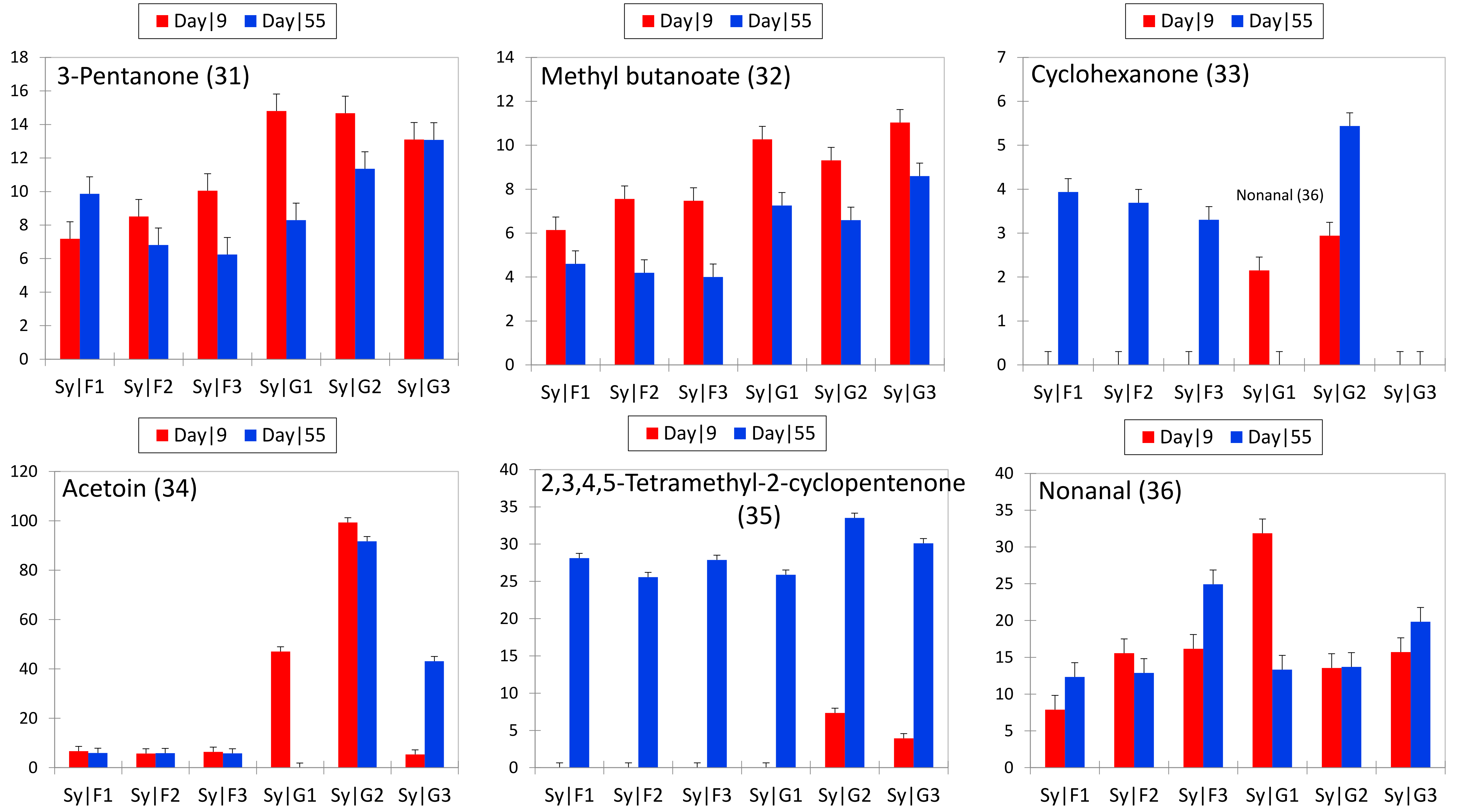
Day|9 Day|55
3-Pentanone (31)
Methyl butanoate (32)
Cyclohexanone (33)
Nonanal (36)
Acetoin (34)
2,3,4,5-Tetramethyl-2-cyclopentenone (35)
Nonanal (36)
Sy|F1 Sy|F2 Sy|F3 Sy|G1 Sy|G2 Sy|G3
0 2 4 6 8 10 12 14 16 18
0 1 2 3 4 5 6 7
0 20 40 60 80 100 120
0 5 10 15 20 25 30 35 40

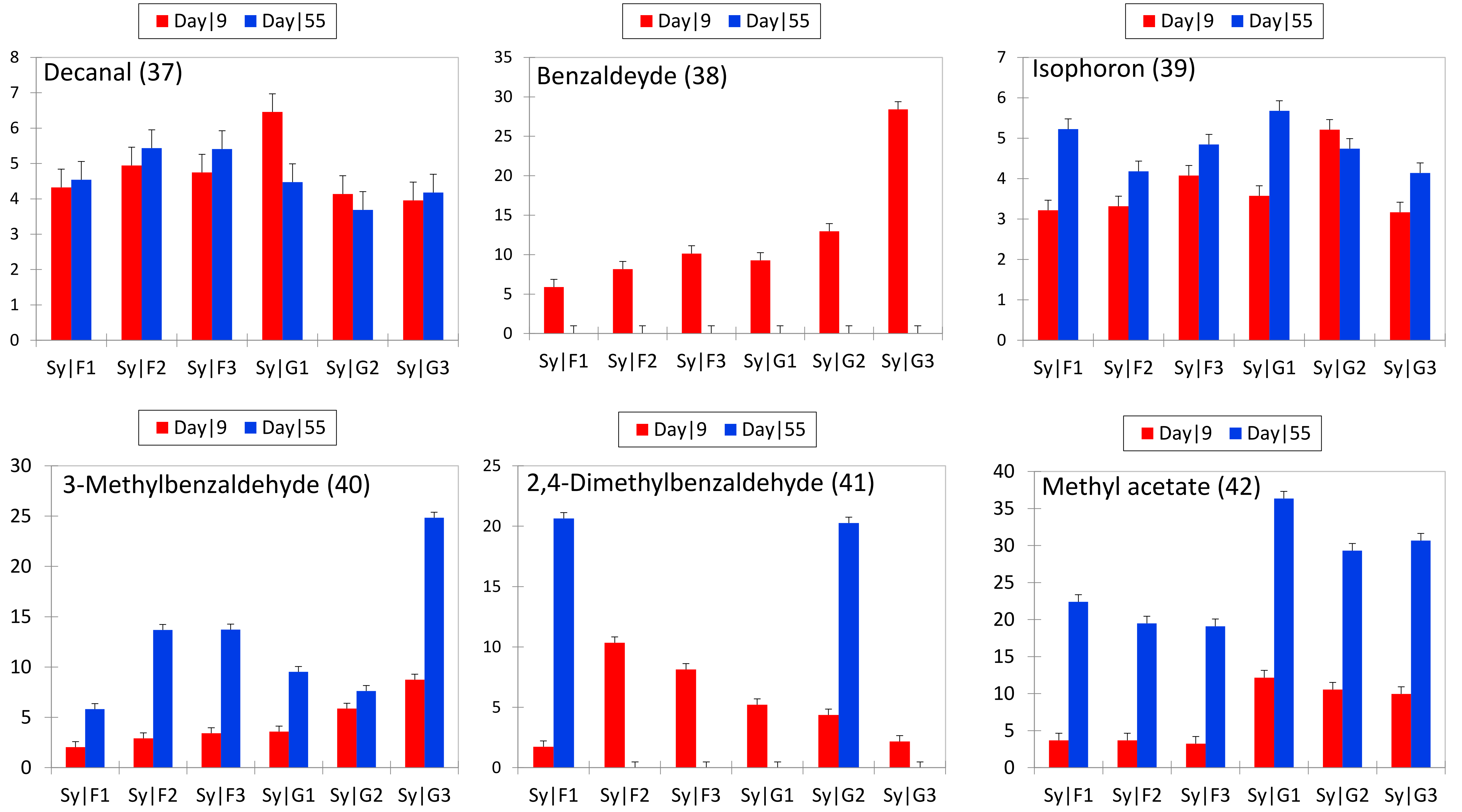
Day|9
Day|55
Decanal (37)
Benzaldeyde (38)
Isophoron (39)
3-Methylbenzaldehyde (40)
2,4-Dimethylbenzaldehyde (41)
Methyl acetate (42)
Sy|F1
Sy|F2
Sy|F3
Sy|G1
Sy|G2
Sy|G3

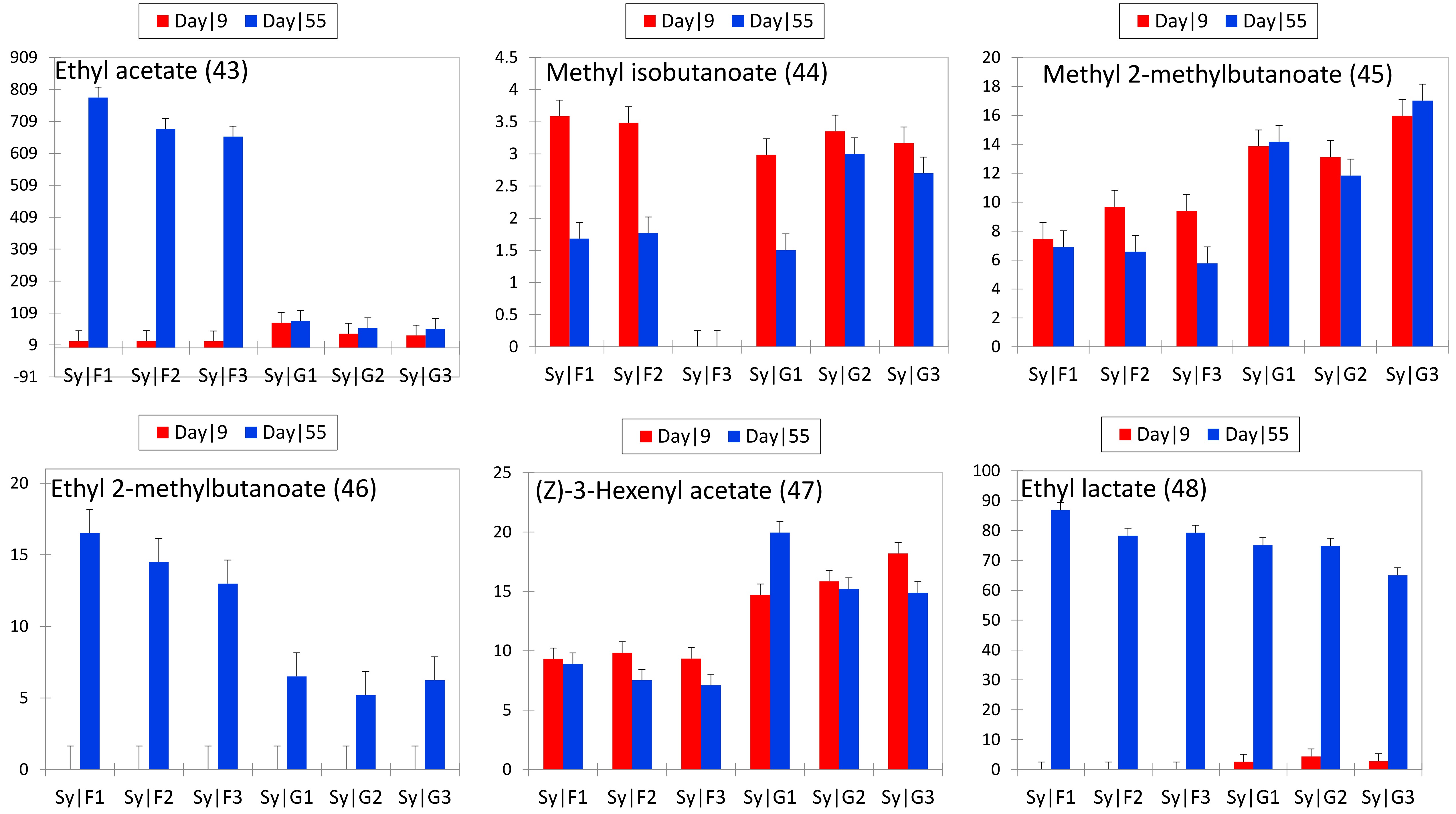
Ethyl acetate (43)
Methyl isobutanoate (44)
Methyl 2-methylbutanoate (45)
Ethyl 2-methylbutanoate (46)
(Z)-3-Hexenyl acetate (47)
Ethyl lactate (48)
Day|9
Day|55
Sy|F1
Sy|F2
Sy|F3
Sy|G1
Sy|G2
Sy|G3

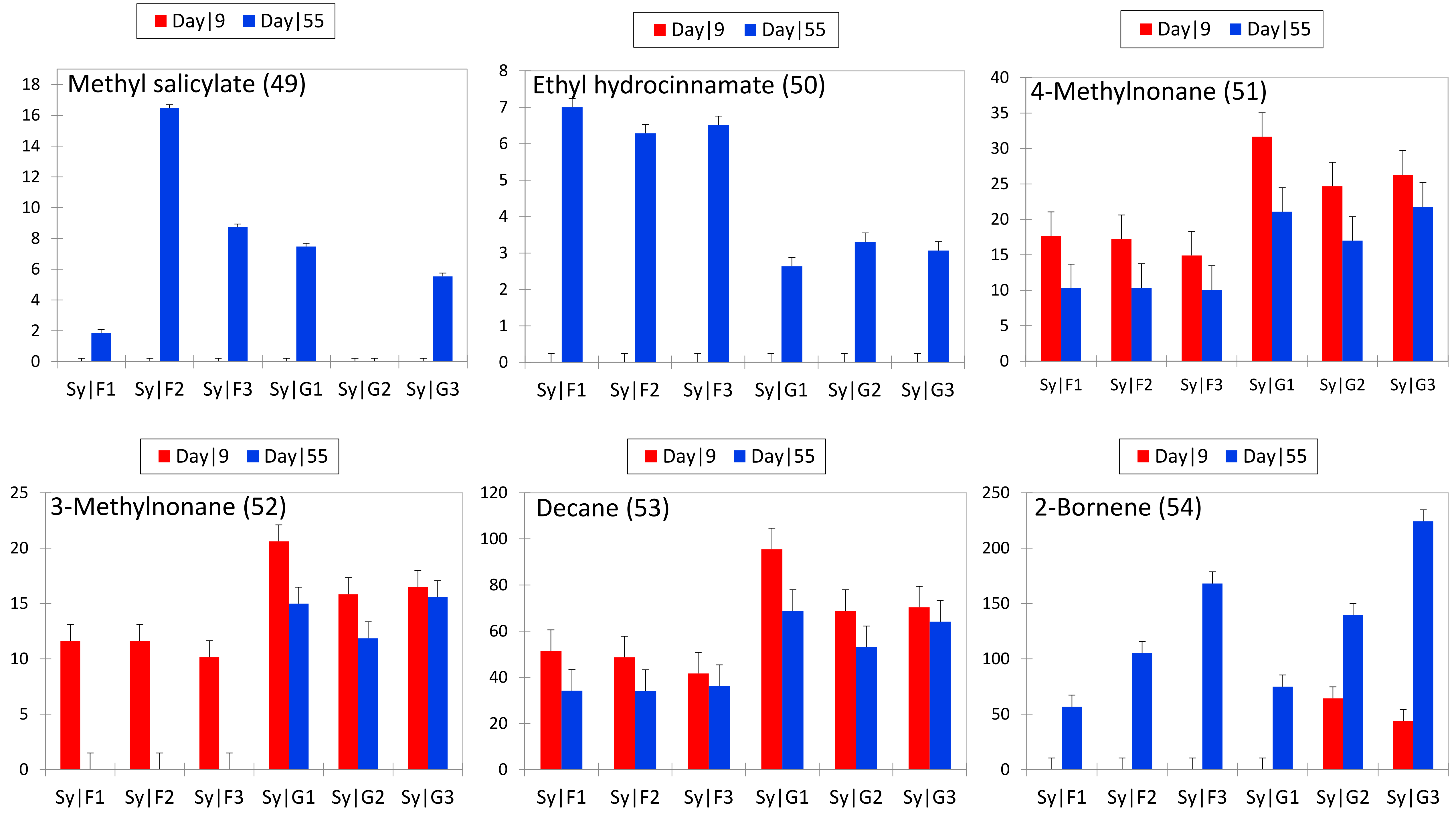

Day|9
Day|55
Methyl salicylate (49)
18
16
14
12
10
8
6
4
2
0
Sy|F1
Sy|F2
Sy|F3
Sy|G1
Sy|G2
Sy|G3
Ethyl hydrocinnamate (50)
8
7
6
5
4
3
2
1
0
4-Methylnonane (51)
40
35
30
25
20
15
10
5
0
3-Methylnonane (52)
25
20
15
10
5
0
Decane (53)
120
100
80
60
40
20
0
2-Bornene (54)
250
200
150
100
50
0

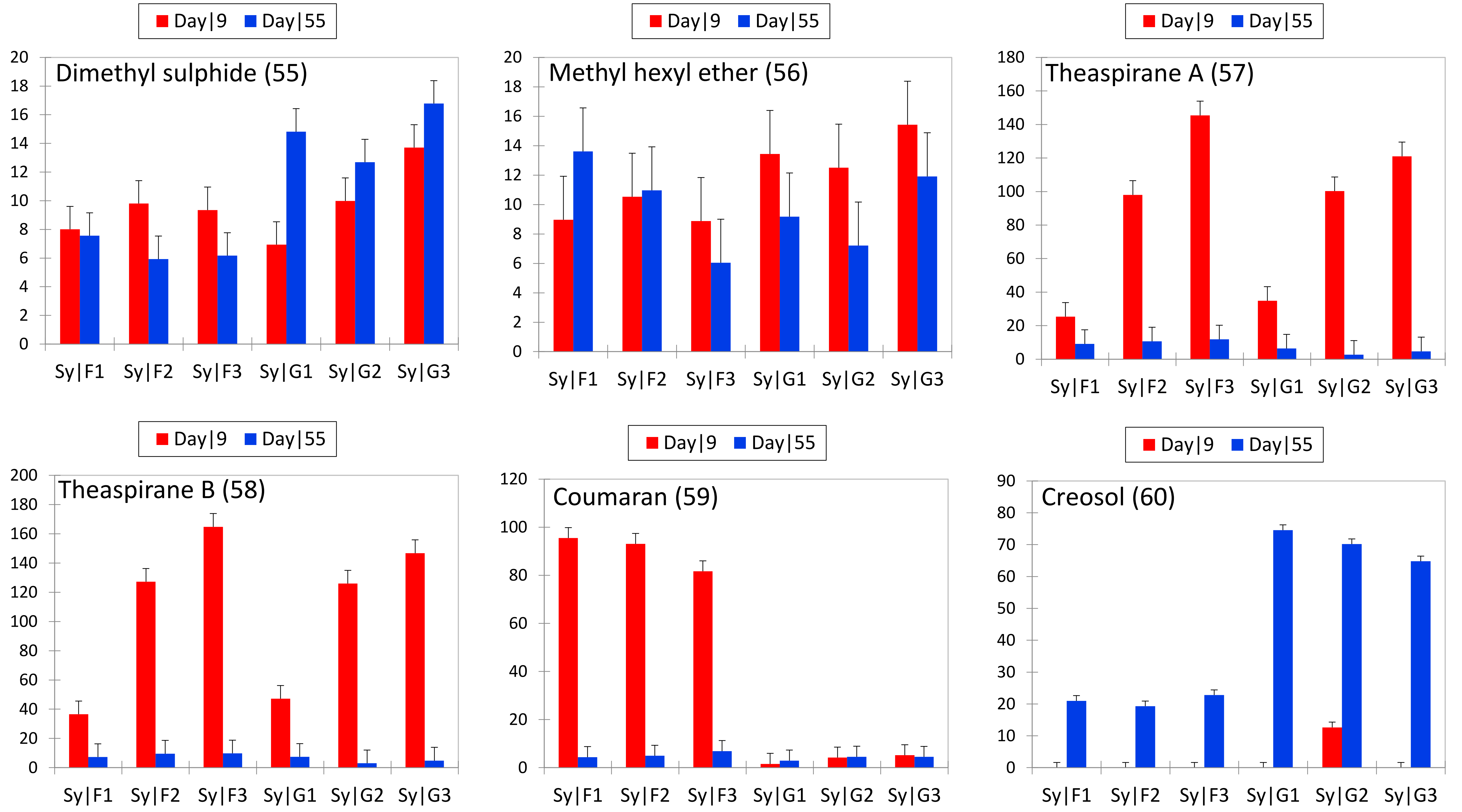
Day|9
Day|55
Dimethyl sulphide (55)
0 2 4 6 8 10 12 14 16 18 20
Sy|F1 Sy|F2 Sy|F3 Sy|G1 Sy|G2 Sy|G3
Day|9
Day|55
Methyl hexyl ether (56)
0 2 4 6 8 10 12 14 16 18 20
Sy|F1 Sy|F2 Sy|F3 Sy|G1 Sy|G2 Sy|G3
Day|9
Day|55
Theaspirane A (57)
0 20 40 60 80 100 120 140 160 180
Sy|F1 Sy|F2 Sy|F3 Sy|G1 Sy|G2 Sy|G3
Day|9
Day|55
Theaspirane B (58)
0 20 40 60 80 100 120 140 160 180 200
Sy|F1 Sy|F2 Sy|F3 Sy|G1 Sy|G2 Sy|G3
Day|9
Day|55
Coumaran (59)
0 20 40 60 80 100 120
Sy|F1 Sy|F2 Sy|F3 Sy|G1 Sy|G2 Sy|G3
Day|9
Day|55
Creosol (60)
0 10 20 30 40 50 60 70 80 90
Sy|F1 Sy|F2 Sy|F3 Sy|G1 Sy|G2 Sy|G3

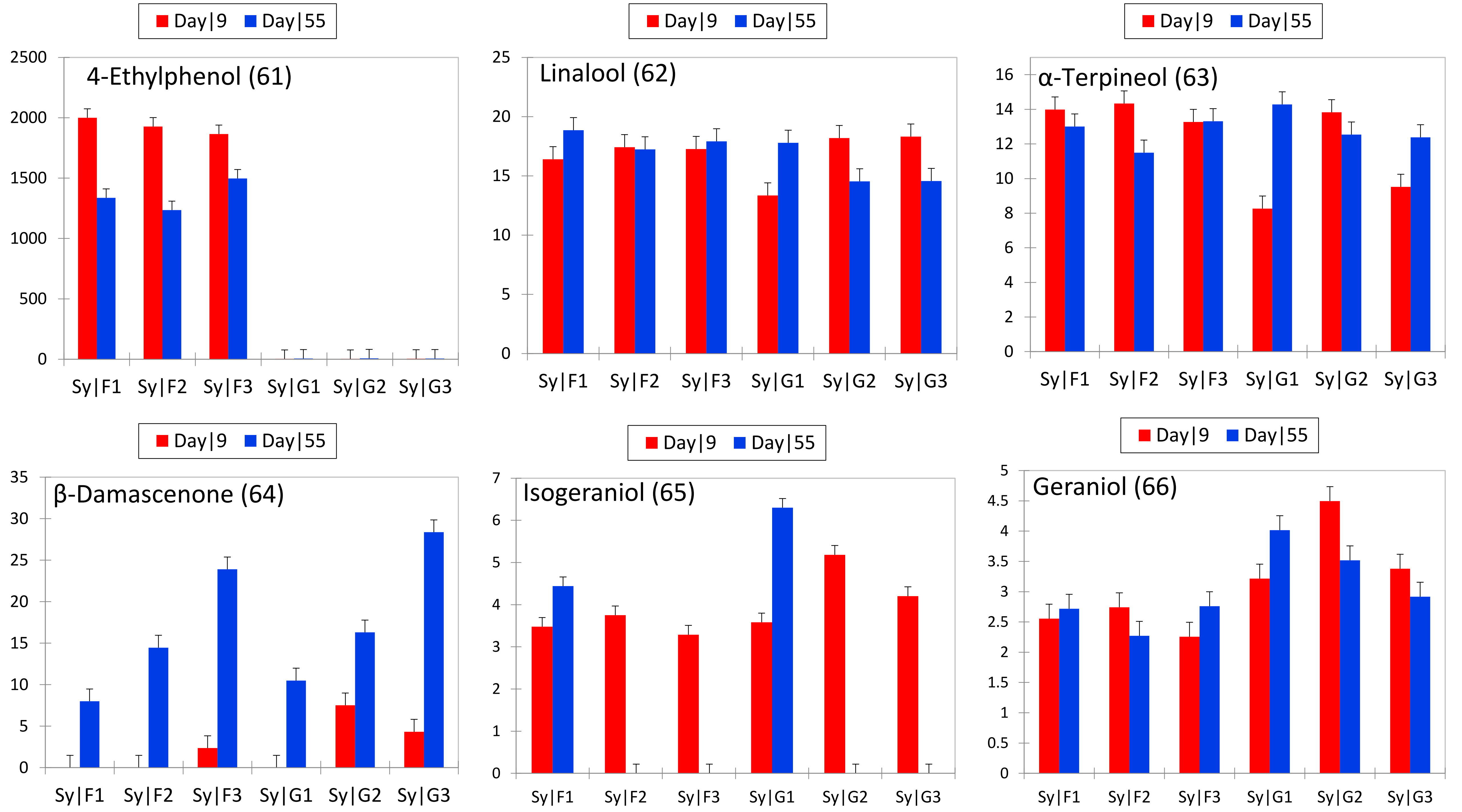
Day|9 Day|55
4-Ethylphenol (61)
2500
2000
1500
1000
500
0
Sy|F1 Sy|F2 Sy|F3 Sy|G1 Sy|G2 Sy|G3
Day|9 Day|55
Linalool (62)
25
20
15
10
5
0
Sy|F1 Sy|F2 Sy|F3 Sy|G1 Sy|G2 Sy|G3
Day|9 Day|55
α-Terpineol (63)
16
14
12
10
8
6
4
2
0
Sy|F1 Sy|F2 Sy|F3 Sy|G1 Sy|G2 Sy|G3
Day|9 Day|55
β-Damascenone (64)
35
30
25
20
15
10
5
0
Sy|F1 Sy|F2 Sy|F3 Sy|G1 Sy|G2 Sy|G3
Day|9 Day|55
Isogeraniol (65)
7
6
5
4
3
2
1
0
Sy|F1 Sy|F2 Sy|F3 Sy|G1 Sy|G2 Sy|G3
Day|9 Day|55
Geraniol (66)
5
4.5
4
3.5
3
2.5
2
1.5
1
0.5
0
Sy|F1 Sy|F2 Sy|F3 Sy|G1 Sy|G2 Sy|G3

Figure S1. Mean and standard deviations of common volatile organic compounds (VOCS) in the Spanish-style Manzanilla cultivar, processed in interconnected under-vacuum fermenters (F system) and traditional independent fermenters (G system). The graph illustrates patterns for each fermenter within each process at the two fermentation periods. Values were obtained from ANOVA analysis. Compounds are numbered in parentheses for reference in the text.
