# Supplementary material for: Volatile Dynamics and Their Associations with Microbial Genera in Spanish-Style Table Olives Processed in Interconnected Under-Vacuum and Fiberglass Independent Vessels
Source: Foods. 2026 Aug 27;15(17):3034. doi: 10.3390/foods15173034 (PMC13565360; doi:10.3390/foods15173034)
Supplement: Supplementary file 1 [file foods-15-03034-s001.zip › Figure S2.pdf]

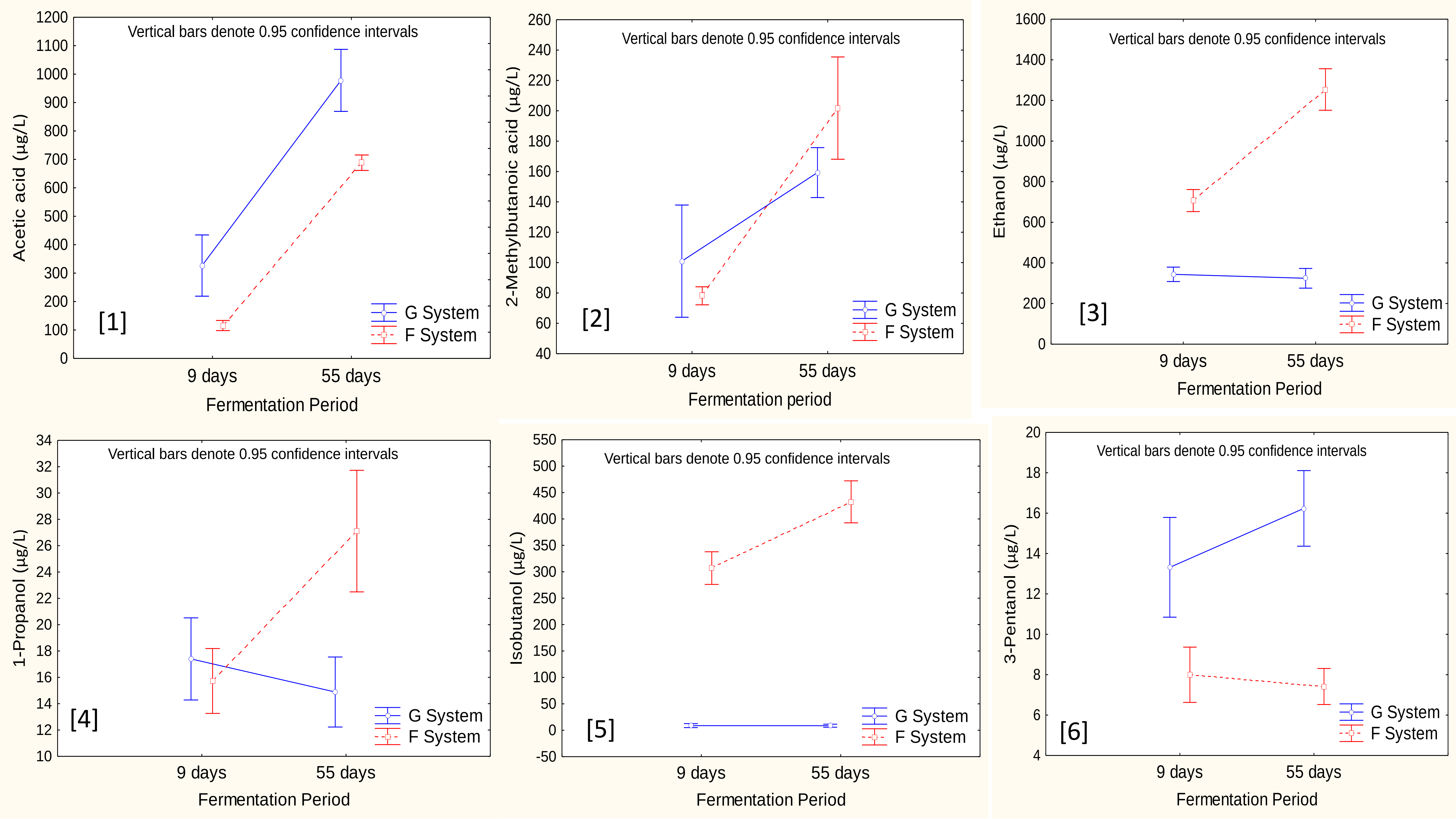

Vertical bars denote 0.95 confidence intervals
Acetic acid (µg/L)
1200
1100
1000
900
800
700
600
500
400
300
200
100
0
[1]
G System
F System
9 days
55 days
Fermentation Period
Vertical bars denote 0.95 confidence intervals
2-Methylbutanoic acid (µg/L)
260
240
220
200
180
160
140
120
100
80
60
40
[2]
G System
F System
9 days
55 days
Fermentation period
Vertical bars denote 0.95 confidence intervals
Ethanol (µg/L)
1600
1400
1200
1000
800
600
400
200
0
[3]
G System
F System
9 days
55 days
Fermentation Period
Vertical bars denote 0.95 confidence intervals
1-Propanol (µg/L)
34
32
30
28
26
24
22
20
18
16
14
12
10
[4]
G System
F System
9 days
55 days
Fermentation Period
Vertical bars denote 0.95 confidence intervals
Isobutanol (µg/L)
550
500
450
400
350
300
250
200
150
100
50
0
-50
[5]
G System
F System
9 days
55 days
Fermentation Period
Vertical bars denote 0.95 confidence intervals
3-Pentanol (µg/L)
20
18
16
14
12
10
8
6
4
[6]
G System
F System
9 days
55 days
Fermentation Period

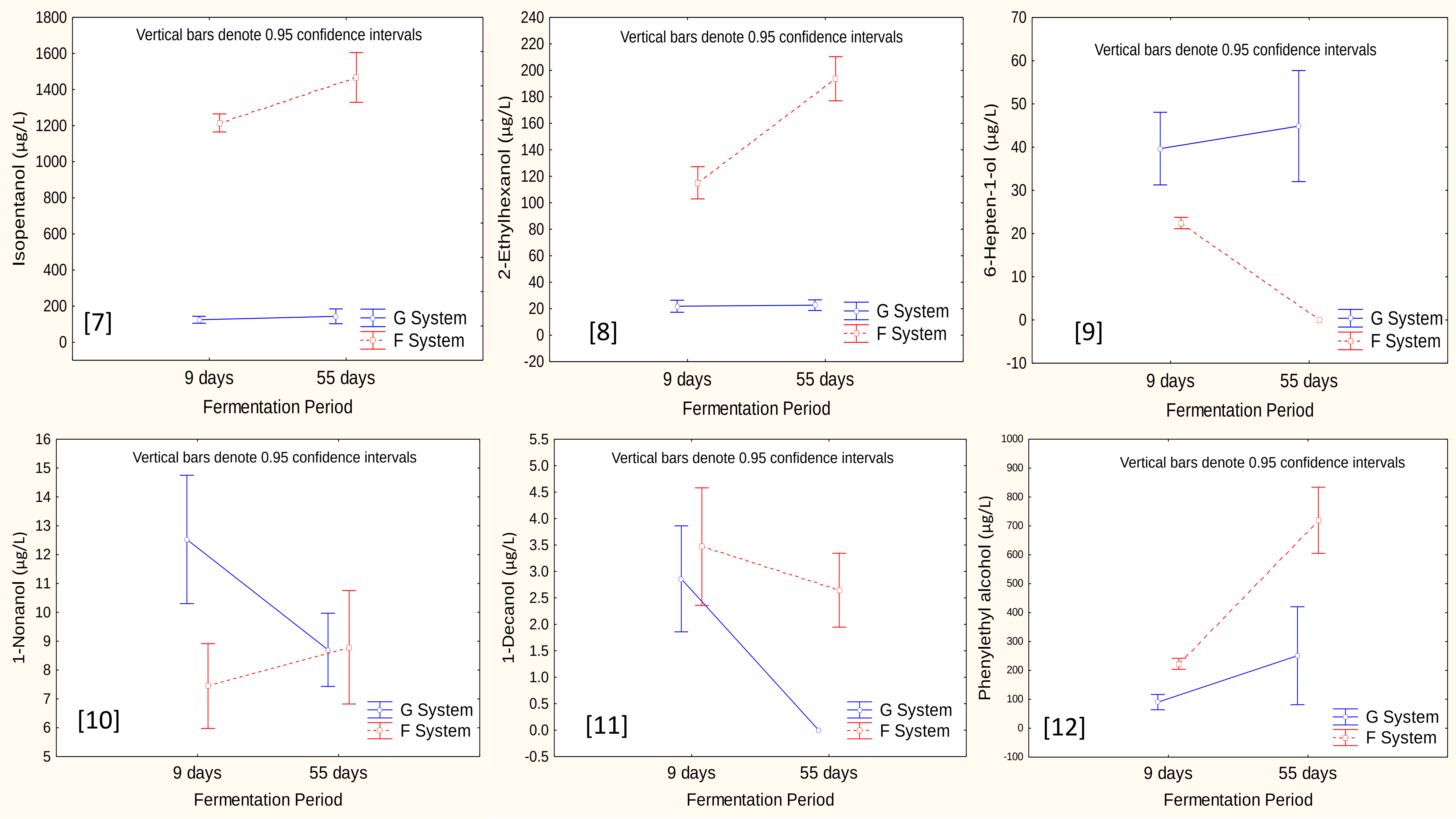
Vertical bars denote 0.95 confidence intervals
Isopentanol (µg/L)
1800
1600
1400
1200
1000
800
600
400
200
0
[7]
G System
F System
9 days
55 days
Fermentation Period
Vertical bars denote 0.95 confidence intervals
2-Ethylhexanol (µg/L)
240
220
200
180
160
140
120
100
80
60
40
20
0
-20
[8]
G System
F System
9 days
55 days
Fermentation Period
Vertical bars denote 0.95 confidence intervals
6-Hepten-1-ol (µg/L)
70
60
50
40
30
20
10
0
-10
[9]
G System
F System
9 days
55 days
Fermentation Period
Vertical bars denote 0.95 confidence intervals
1-Nonanol (µg/L)
16
15
14
13
12
11
10
9
8
7
6
5
[10]
G System
F System
9 days
55 days
Fermentation Period
Vertical bars denote 0.95 confidence intervals
1-Decanol (µg/L)
5.5
5.0
4.5
4.0
3.5
3.0
2.5
2.0
1.5
1.0
0.5
0.0
-0.5
[11]
G System
F System
9 days
55 days
Fermentation Period
Vertical bars denote 0.95 confidence intervals
Phenylethyl alcohol (µg/L)
1000
900
800
700
600
500
400
300
200
100
0
-100
[12]
G System
F System
9 days
55 days
Fermentation Period

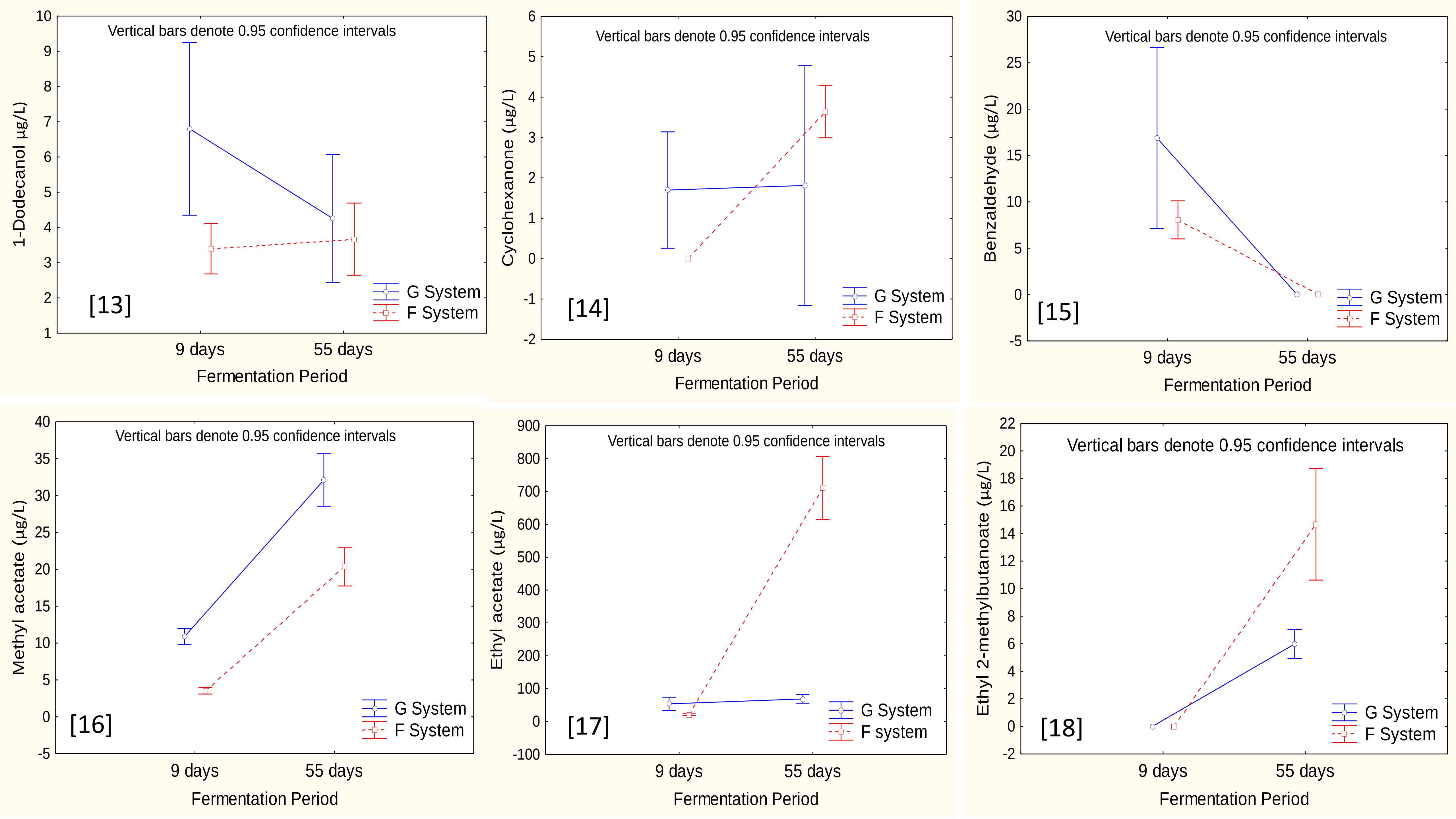
Vertical bars denote 0.95 confidence intervals
1-Dodecanol (μg/L)
[13]
G System
F System
9 days
55 days
Fermentation Period
Vertical bars denote 0.95 confidence intervals
Cyclohexanone (μg/L)
[14]
G System
F System
9 days
55 days
Fermentation Period
Vertical bars denote 0.95 confidence intervals
Benzaldehyde (μg/L)
[15]
G System
F System
9 days
55 days
Fermentation Period
Vertical bars denote 0.95 confidence intervals
Methyl acetate (μg/L)
[16]
G System
F System
9 days
55 days
Fermentation Period
Vertical bars denote 0.95 confidence intervals
Ethyl acetate (μg/L)
[17]
G System
F system
9 days
55 days
Fermentation Period
Vertical bars denote 0.95 confidence intervals
Ethyl 2-methylbutanoate (μg/L)
[18]
G System
F System
9 days
55 days
Fermentation Period

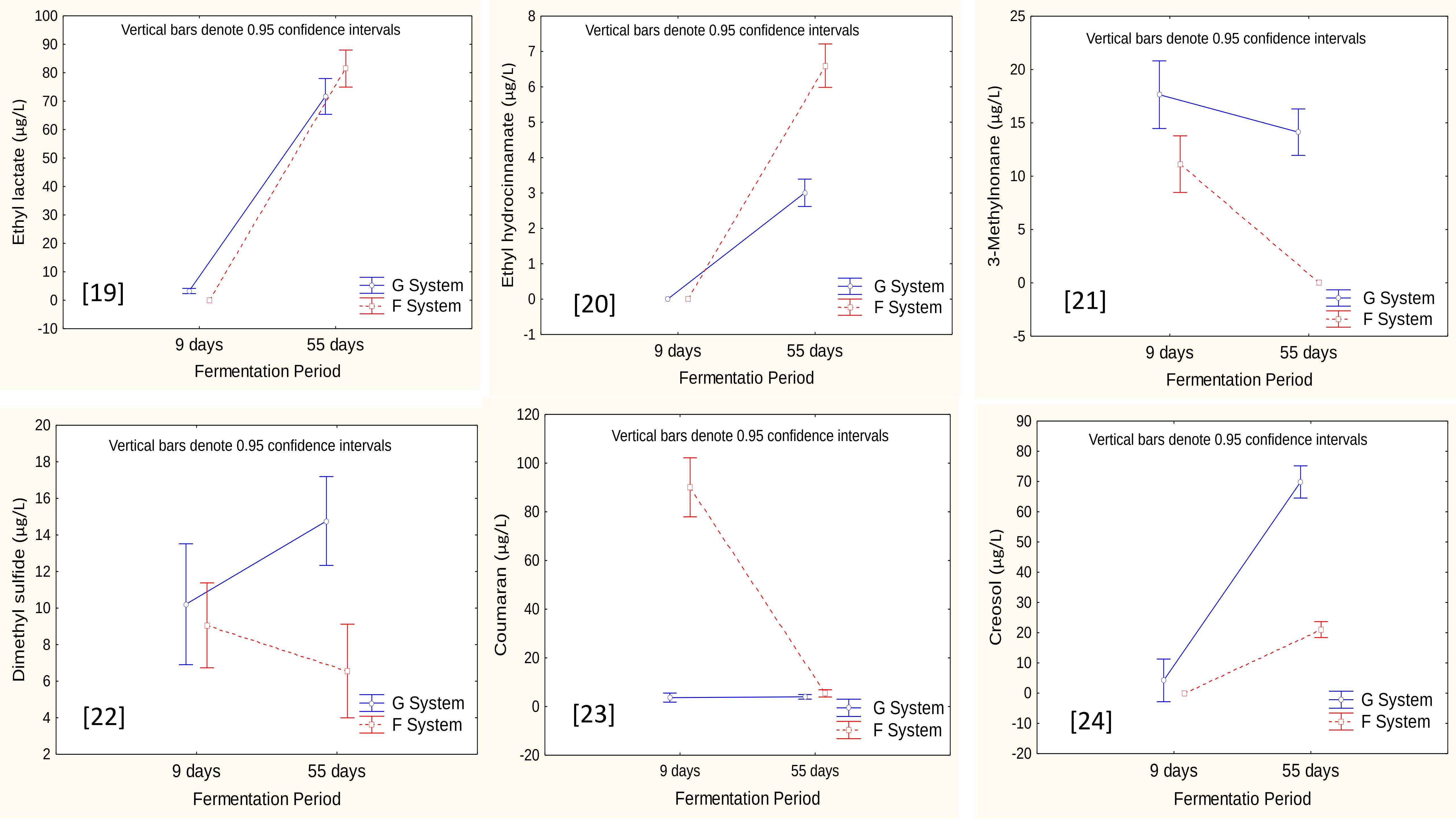
Vertical bars denote 0.95 confidence intervals
Ethyl lactate (µg/L)
100
90
80
70
60
50
40
30
20
10
0
-10
[19]
G System
F System
9 days
55 days
Fermentation Period
Vertical bars denote 0.95 confidence intervals
Ethyl hydrocinnamate (µg/L)
8
7
6
5
4
3
2
1
0
-1
[20]
G System
F System
9 days
55 days
Fermentatio Period
Vertical bars denote 0.95 confidence intervals
3-Methylnonane (µg/L)
25
20
15
10
5
0
-5
[21]
G System
F System
9 days
55 days
Fermentation Period
Vertical bars denote 0.95 confidence intervals
Dimethyl sulfide (µg/L)
20
18
16
14
12
10
8
6
4
2
[22]
G System
F System
9 days
55 days
Fermentation Period
Vertical bars denote 0.95 confidence intervals
Coumaran (µg/L)
120
100
80
60
40
20
0
-20
[23]
G System
F System
9 days
55 days
Fermentation Period
Vertical bars denote 0.95 confidence intervals
Creosol (µg/L)
90
80
70
60
50
40
30
20
10
0
-10
-20
[24]
G System
F System
9 days
55 days
Fermentatio Period

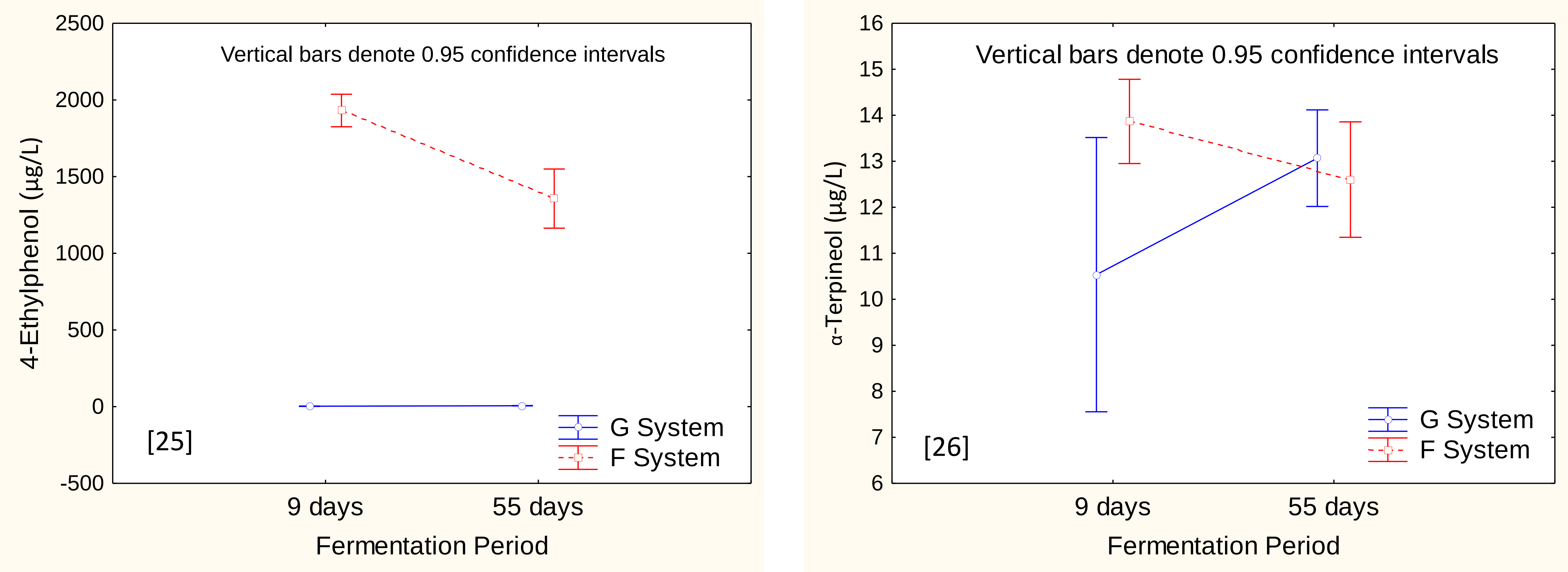

Figure S2. Mean values and confidence limits of volatile organic compounds (VOCs) in the Spanish-style Manzanilla cultivar showing significant interactions between fermentation systems (F and G) and fermentation periods. The graph illustrates overall patterns for each system and fermentation periods. Values were obtained from ANOVA analysis. Compounds are numbered in brackets for reference in the text.
